# Colonization of North America Boosted the Diversification of Whiptail Lizards

**DOI:** 10.1002/ece3.70418

**Published:** 2024-10-23

**Authors:** Humberto Coelho Nappo, Guarino Rinaldi Colli

**Affiliations:** ^1^ Programa de Pós‐Graduação em Ecologia, Instituto de Ciências Biológicas Universidade de Brasília Brasília DF Brazil; ^2^ Departamento de Zoologia, Instituto de Ciências Biológicas Universidade de Brasília Brasília DF Brazil

**Keywords:** diversification analysis, ecological niche overlap, habitat availability, state‐dependent speciation‐extinction, Teiidae

## Abstract

Diversification is frequently associated with change—anything from colonizing a new area to evolving a new trait. Once a lineage changes, the organisms may be able to exploit previously unavailable ecological opportunities and release pressures from predators, parasites, and competitors, which may increase the speciation rate. Modern teiid lizards originated in South America but managed to colonize and diversify in North America. We assessed whether geographic distribution, body size, and body temperatures are associated with teiid diversification using GeoHiSSE and inverse equal‐splits statistics with simulation tests. We also estimated speciation rates with MiSSE to account for the effect of unmeasured variables. Moreover, we assessed the ecological niche overlap between North American (including Caribbean) teiids and their sister clade in South America. Our results indicate that only distribution range affected diversification, but we discuss that the available data might not have been enough to assess the effect of body temperatures. We also show that North American teiids have a broader ecological niche encompassing almost all environmental conditions used by their sister clade in South America but expanding mainly toward arid areas. Our results suggest that this expansion significantly impacted teiid diversification due to the seizing of ecological opportunities or ecological release, but we do not discard possible effects of phenotypic evolution.

## Graphical abstract

Teiids increased their diversification rates upon colonization of North America, which was associated with an ecological niche expansion toward drier areas. This increment in diversification was not shown to be associated with phenotypic traits, which suggests it was due to ecological opportunity and/or ecological release.

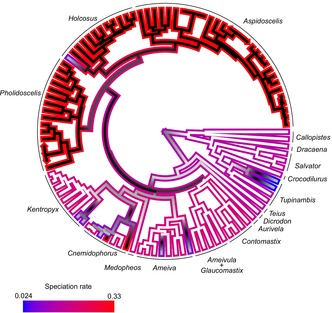

## 1 Introduction

Phenotypic evolution is frequently evoked to explain diversification, even though the link may be context‐dependent and, thus, not so easily identified (de Queiroz 2002). Multiple studies have already reported shifts in diversification rates associated with the emergence of traits, which include morphological, physiological, ecological, and behavioral characteristics (e.g., Biffin et al. 2010; Laserna and Herrera 2017; Wessinger, Rausher, and Hilema 2019). However, the same has also been observed for external changes such as habitat shifts or colonization of new areas (Ballarin and Li 2018; Hou et al. 2011).

Colonizing a new habitat enables the organisms to explore ecological opportunities—resources underused by other taxa—and escape predators, competitors, and parasites (Schluter 2000). This favorable scenario enables the expansion of niche breadth (Gillespie et al. 2020). Posteriorly, divergent selection acting upon populations in different environments generates multiple specialized lineages that differentiate morphologically and ecologically from each other, constituting adaptive radiation (Gillespie et al. 2020; Schluter 2000). Indeed, the main drivers of diversification in squamates were range area expansion and arboreality—two characteristics associated with colonizing new habitats (Li and Wiens 2022).

Squamates are good candidates to show phenotypic evolution on physiological grounds associated with the colonization of new habitats thanks to their high dependence on environmental temperatures. Lizards are ectotherms and environmental temperatures directly influence their body temperatures, which play a crucial role in the colonization and persistence in novel environments (Lanna et al. 2022). Since these temperatures are associated with different types of habitats (Lanna et al. 2022), changes in body temperatures could be associated with habitat shifts and, therefore, diversification shifts. Body size has been long suggested as a predictor for diversification, based on the idea that smaller organisms may have shorter generation times and, thus, speciate faster (Hutchinson and Macarthur 1959). However, this trait does not seem relevant for squamates (Feldman et al. 2016; Li and Wiens 2022). Nonetheless, patterns observed at such a large phylogenetic scale do not necessarily carry on to nested clades since the processes affecting ecology, distribution, and diversification vary at all scales (Graham, Storch, and Machac 2018). For instance, body size significantly affected snake diversification, which could not be found for squamates as a whole (Feldman et al. 2016). Thus, testing these and other predictors in more restricted phylogenetic scales may reveal specific trait–diversification relationships.

An exciting group to test the drivers of diversification is the lizard family Teiidae Gray, 1827, which is composed of whiptails, racerunners, and tegus. Fossil evidence suggests that the most recent ancestor of Teiidae + Gymnophthalmidae originated in North America (Nydam, Eaton, and Sankey 2007). Nonetheless, molecular phylogenies show that all living teiids trace their history to a South American ancestor (Giugliano, Collevatti, and Colli 2007). These South American ancestors managed to disperse northwards and reached North America and the Antilles as early as the Miocene, as shown by fossil data and ancestral area reconstruction (Scarpetta 2020; Tucker et al. 2017). Although most northern teiids descend from this single radiation, some scattered species managed to colonize areas in Central America and Caribbean islands more recently. This family is considerably conservative in ecology, with few scattered species deviating from the heliophilous, active forager, terricolous, and generalist predator pattern (Vitt and Caldwell 2014). However, there is a notorious disparity in species richness and body size between the two teiid subfamilies: Tupinambinae Bonaparte, 1831 and Teiinae Gray, 1827. While the former is composed of only 16 large‐bodied tegu species (mean maximum snout–vent length (SVL) = 379.1 ± 121.7 mm; range = 173–614 mm), the latter has over 150 smaller‐bodied whiptail and racerunner species (mean max SVL = 112.9 ± 37.79 mm; range = 52.4–250 mm; Meiri et al. 2018). The bulk of the diversity within the family is carried by the cnemidophorines, a clade composed of all Teiinae to the exclusion of *Teius* and *Dicrodon* (Reeder, Cole, and Dessauer 2002).

Whereas the hypothetical correlation between body size and diversification in teiids seems evident, a less conspicuous association could also link physiology and diversification. Teiidae maximum active body temperatures oscillate from low 30s to low 40s (°C), with the highest maximums within the desert‐dwelling *Aspidoscelis* (Meiri et al. 2018, 2013)—the most diverse genus in the family. The lower maximum temperatures, on the other hand, belong to species in less diverse genera, such as *Crocodilurus*, *Dracaena*, *Dicrodon*, and *Kentropyx*. However, habitat significantly affects body temperatures, and semi‐aquatic, arboreal, or forest‐dwelling lineages, such as the ones cited above, are expected to attain lower temperatures. Therefore, a correlation between diversification rates and body temperature could result from an effect of habitat instead of a direct relationship between temperature and diversification.

Notwithstanding, if phenotype is conserved, diversification shifts could result from changes extrinsic to the organisms. This should happen if a lineage was preadapted to the colonized area or if the new area is environmentally similar to its center of origin. Since no novel adaptation would be needed, descendants from these migrants should have very similar ecological requirements to those of their closest relatives. When this is the case, we should expect not to find any correlation between traits and diversification and observe high niche overlap between immigrants and their non‐migrant relatives.

This study aims to assess whether diversification in Teiidae is associated with body size, body temperatures, and distribution range and to assess ecological niche overlap between North American (including Caribbean) teiids and its sister clade in South America. Since the colonization of new areas is frequently associated with increased diversification, we expect to observe higher diversification rates associated with a North American distribution. For phenotypic traits, we expect higher diversification rates to be associated with smaller sizes and higher body temperatures. Alternatively, if temperature is unrelated to diversification, this should mean that teiids did not need extensive physiological adaptations to colonize North America—either because the conditions in the new continent were similar to South America or because of preadaptation. In either case, we expect to find North American teiids occupying niches similar to those of their southern closest relatives.

## 2 Materials and Methods

### 2.1 Data Collection

We gathered data for maximum SVL, average minimum, average, and average maximum body temperature of active lizards, mostly from Global Assessment of Reptile Distributions (GARD) publications (Caetano et al. 2022; Meiri et al. 2018, 2013; Roll et al. 2017) and complemented it with data published in later studies. All trait data and the corresponding references are in File S1.

To assess ecological niche overlap, we downloaded two sets of occurrence records from the Global Biodiversity Information Facility (GBIF) (GBIF.org 2023): one for the migrant clade, composed of *Aspidoscelis*, *Holcosus* and *Pholidoscelis*, and one for its South American sister clade composed of *Ameiva*, *Cnemidophorus*, *Kentropyx* and *Medopheos*. We only used records associated with preserved specimens from identified institutions after 2000, with individuals' counts greater than 0. We also used the *clean_coordinates()* function from the “CoordinateCleaner” package, version 2.0.20 (Zizka et al. 2019) to identify and exclude potentially erroneous records as coordinates falling in the sea or inside urban areas, coinciding with countries' centroids, next to biodiversity research institutions and other common spatial errors. Then, we generated random pseudoabsences for each clade in the corresponding continent using the *spatSample()* function from the “terra” package version 1.7.18 (Hijmans et al. 2023). We downloaded elevation and bioclimatic data from the Shuttle Radar Topography Mission and WorldClim 2.1 (Fick and Hijmans 2017; Jarvis et al. 2008) at a resolution of 2.5 min. We also downloaded the normalized difference vegetation index (NDVI) at a resolution of 0.05° from the Terra satellite at EarthData (Didan and Huete 2015). The NDVI is the difference between near‐infrared and visible spectral reflectance divided by their sum. This index is directly correlated with photosynthetic capacity and can be used to characterize the environment. We downloaded monthly NDVI data from January 1, 2001 to December 31, 2020, and calculated mean values for each cell across all rasters. Since NDVI data are unavailable for high latitudes, we used the mean NDVI raster as a mask for transforming elevation and bioclimatic rasters to avoid dealing with empty cells. This approach should not impact our analyses since the maximum latitude limit for this data is way beyond the teiid distribution range. Then, we removed variables with a variance inflation factor (VIF) greater than 3.5 and extracted the values of each remaining variable for both presence and pseudoabsence coordinates.

### 2.2 Trait‐Dependent Diversification

We updated teiid sequences in the multiple sequence alignment from Tonini et al. (2016) and ran a constrained tree search in IQ‐TREE, version 2.2.2.6 (Minh et al. 2020) using the topology from Tucker et al. (2016) as a monophyletic constraint. Then, we ran a divergence time analysis in MEGA11 using the RelTime method (Tamura, Stecher, and Kumar 2021; Tamura, Tao, and Kumar 2018). We used two fossils to calibrate the timetree: one at the base of *Callopistes* and another at the base of cnemidophorines (Quadros, Chafrat, and Zaher 2018; Scarpetta 2020). The final tree included 106 teiid species, approximately 60% of known diversity. More detailed explanations for the phylogenetic inference may be found in Appendix A, and the resulting tree file we used in the diversification analyses is in File S2.

We assessed whether diversification was associated with distribution range using GeoHiSSE, a modification of GeoSSE (Caetano, O'Meara, and Beaulieu 2018; Goldberg, Lancaster, and Ree 2011). State‐dependent speciation and extinction models (SSE) are models in which macroevolutionary rates depend on the state of a character. In GeoSSE models, species are allowed to simultaneously have two states of a character (geographic distribution), in which case their rates will correspond to the summation of the effects of both areas. GeoHiSSE essentially expands GeoSSE to account for the effect of unmeasured variables (hidden states). Considering the effect of hidden states is essential in an SSE framework because otherwise, null models become trivial, which inflates the false positive rate (Beaulieu and O'Meara 2016; Caetano, O'Meara, and Beaulieu 2018). We implemented four GeoHiSSE models: range‐independent diversification/without hidden states (1); range‐dependent diversification/without hidden states (2); range‐independent diversification/with hidden states (3); range‐dependent diversification/with hidden states (4). Distribution ranges for each species were classified as “North America” if restricted to Central and North America, including Caribbean islands; “South America” if restricted to mainland South America and islands outside the Caribbean Sea; or “both” if included mainland South America and North America/Caribbean islands. We ran GeoHiSSE models twice, once with the full tree and once after removing eight short‐branched tips (*Pholidoscelis corvinus*, *P. corax*, *Aspidoscelis marmoratus*, *A. tigris*, *A. neotesselatus*, *A. sonorae*, *A. uniparens*, and *A. opatae*) that we believe could affect our results. We accounted for the proportion of sampled species in each area, which were 0.527 (South America), 0.6556 (North America), and 0.8 (both) for the first run and 0.527, 0.5667, and 0.8 for the second. The area matrixes with the classification of the distribution range for each species are in Files S3 and S4.

Since most North American teiids descend from a single radiation, the evolution of a feature that affected diversification within this clade could confound our interpretation of GeoHiSSE results. This could happen because an unmeasured variable affecting the diversification dynamics of a sub‐clade restricted to one of the areas would inflate the effect of the area. To account for this issue, we also estimated macroevolutionary rates with a Missing State Speciation and Extinction model (MiSSE) (Vasconcelos, O'Meara, and Beaulieu 2022). MiSSE models only account for the influence of hidden states and can reveal whether the diversification dynamics matches our hypothesis (whether a diversification burst occurred immediately following the colonization of North America or within a nested clade a long time after it, for example). As we did for GeoHiSSE, we fitted MiSSE models twice to our tree. The first time we used our full tree considering a global sampling fraction of 61%, and the second time, we adjusted the sampling fraction to 56% for the pruned tree. We decided to rerun the analysis without those species because the extremely short branch lengths—probably due to hybridization—significantly affected MiSSE speciation rate estimates. We tested several parameter combinations for turnover and extinction fraction, limiting the maximum number of parameters to 11 in both runs. We implemented GeoHiSSE and MiSSE analyses through the “hisse” package, version 2.1.11 (Beaulieu and O'Meara 2016).

We used inverse equal‐splits statistics and simulation‐based significance tests (ES‐sim) to assess whether speciation rates were associated with body size and body temperature (Harvey and Rabosky 2018). The inverse equal‐splits metric can be calculated through:

$${\mathrm{ES}}_{i}=\sum_{j=1}^{N_{i}}l_{j}\frac{1}{2^{j-1}}$$

ES_*i*_ corresponds to the diversification rate of tip *i*, *N*_*i*_ is the number of branches between tip *i* and the root, and *l*_*j*_ is the length of each branch *j* from the terminal branch to the root branch (Redding and Mooers 2006). Then, we used ES_*i*_ and trait values to calculate Pearson's correlation coefficient, which we compared to the correlation coefficients of null simulations to assess significance. This method has similar statistical power to QuaSSE but with a lower false positive ratio (Harvey and Rabosky 2018). ES‐sim analysis does not handle missing trait data. Because of this, we ran ES‐sim analyses twice for each trait. Once removing from the tree the species for which we had missing trait data and once with the full tree and imputed trait data. We imputed trait values accounting for tree branch lengths with the *phylopars()* function from the “Rphylopars” R package (Goolsby, Bruggeman, and Ané 2017).

### 2.3 Niche Overlap

We used environmental data from presence/pseudoabsence points for quantifying the ecological niche of the North American lineage and its sister group in South America using an ordination technique recommended by Broennimann et al. (2012). Assessing niche overlap through ordination methods is preferable to using habitat suitability values from species distribution models (SDMs) because the projection of SDMs on another area could lead to unauthentic patterns due to collinearity between biologically relevant and irrelevant predictors (Broennimann et al. 2012). First, we conducted a principal component analysis (PCA) on the environmental data using the *dudi.pca()* function from “ade4” package version 1.7.22 (Dray and Dufour 2007). Then, we calculated the ecological niche space in the environmental ordination and tested for niche equivalency and similarity (Warren, Glor, and Turelli 2008) using the “ecospat” package, version 3.5 (Di Cola et al. 2017). The niche equivalency test assesses whether two ecological niches are identical by grouping all records, splitting them randomly into two data sets the same size as the original ones, and checking if overlap remains constant through iterations. The niche similarity test, on the other hand, tests whether two niches are more similar than random by shifting the occurrence density in one range and calculating the overlap between the simulation and the observed niches. Since niche equivalency and similarity tests do not test exactly the same thing—equivalency is an extreme case of similarity—applying only one of these alternatives could lead to erroneous conclusions about the study system (Warren, Glor, and Turelli 2008). We compared observed and simulated ecological niches for both tests using Schoener's D overlap statistic, which ranges from 0 (no niche overlap) to 1 (identical niches). We ran each test twice, one for each alternative hypothesis (higher or lower equivalency/similarity), using 1000 replications in each case. We implemented all analyses in R, version 4.3.1 (R Core Team 2023), and the code necessary to replicate our results is in File S5.

## 3 Results

### 3.1 Trait‐Dependent Diversification

**FIGURE 1:**
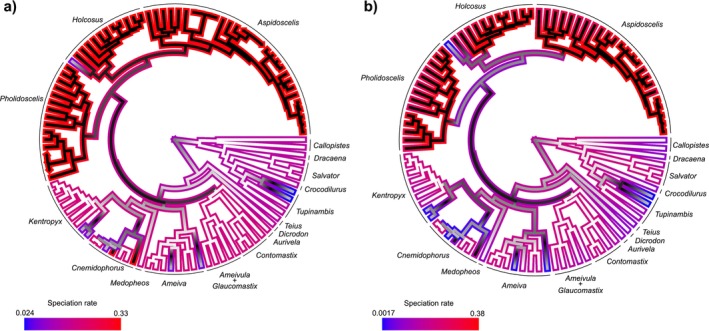
Distribution area and speciation rates estimated for Teiidae by GeoHiSSE for our full Teiidae tree (a) and the same tree without overly short branches (b). Branch colors correspond to the distribution range: White for South America, black for North America, and gray for both. Colors outlining the branches correspond to AIC weighted average speciation rates, following the legend on each inset.

The GeoHiSSE model that best explains teiid diversification for our full tree includes the effect of distribution range without hidden states with a 0.69 AIC weight (Table 1). The second‐best model was the null model with hidden states with a 0.31 AIC weight. The null model without hidden states and the alternative model with hidden states had negligible contributions. For the pruned tree (without short‐branched tips), the best model included both the distribution range and hidden states with an AIC weight of 0.92. Figure 1 depicts diversification rates and distribution ranges for Teiidae species. Rates were estimated using the average of all models weighted by their AIC weights. The average models for both runs attribute higher speciation rates associated with a North American distribution, corroborating our first hypothesis.

**TABLE 1:** AIC weights for GeoHiSSE models with the full tree (middle column) and pruned tree (right column).

Model	AIC weight (full)	AIC weight (pruned)
Range‐independent	2.4 × 10^−5^	5.5 × 10^−6^
Range‐dependent	0.69	5.70 × 10^−2^
Range‐independent + Hidden states	0.31	2.15 × 10^−2^
Range‐dependent + Hidden states	2.8 × 10^−6^	0.92

**FIGURE 2:**
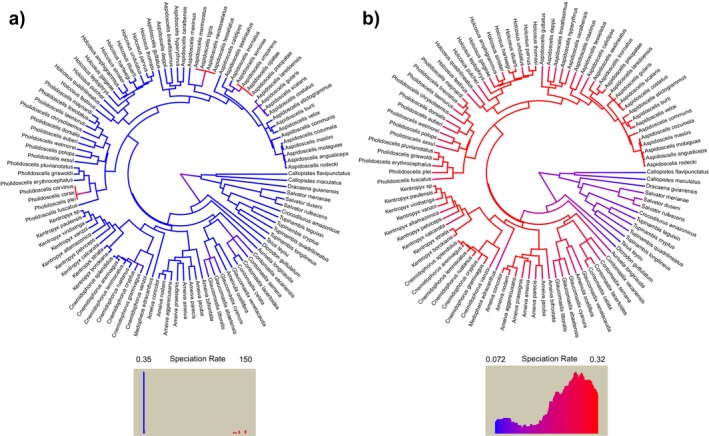
Speciation rates estimated by MiSSE for our full Teiidae tree (a) and the same tree without overly short branches (b). Branch colors correspond to speciation rates following the legend on each inset. The horizontal axis in each inset corresponds to the speciation rate values and the vertical axis corresponds to the frequency of each rate value.

MiSSE analysis with the full tree shows equivalent speciation rates through the tree with three points of outstanding rate increase (Figure 2a). These speciation increments seem to be a product of very short branches likely produced by hybridization. The result of a second run removing some of these short‐branch species is in Figure 2b. After removing these eight species, an increment in speciation rates is observed at the base of cnemidophorines preceding the colonization of North America.

We gathered SVL data for 104 species, average minimum and average maximum active body temperature for 58, and average body temperature for 56. These data and the corresponding references are in File S1. ES‐sim results showed that none of these predictors—either with imputed or unimputed trait data—significantly correlated with diversification rates (Table 2). Our hypotheses were inverse correlation with body size and direct correlation with body temperature.

**TABLE 2:** Pearson's correlation coefficient rho between diversification rates and body size (snout–vent length—SVL), average minimum, average, and average maximum body temperature (Tb) of active teiids, and *p* values for the comparison between observed correlations and simulations.

Predictor	Rho	*p*
Maximum SVL (imputed)	−0.4075660	0.1818182
Average minimum Tb (imputed)	0.2050815	0.5074925
Average Tb (imputed)	0.2688443	0.3976024
Average maximum Tb (imputed)	0.3280758	0.2997003
Maximum SVL	−0.4040589	0.1658342
Average minimum Tb	0.3260446	0.3756244
Average Tb	0.4072709	0.3036963
Average maximum Tb	0.4133281	0.2417582

*Note: p* values in the table do not correspond to the significance level of the correlations between diversification rates and traits. Results are shown for analyses with both imputed and unimputed data.

### 3.2 Niche Overlap

**FIGURE 3:**
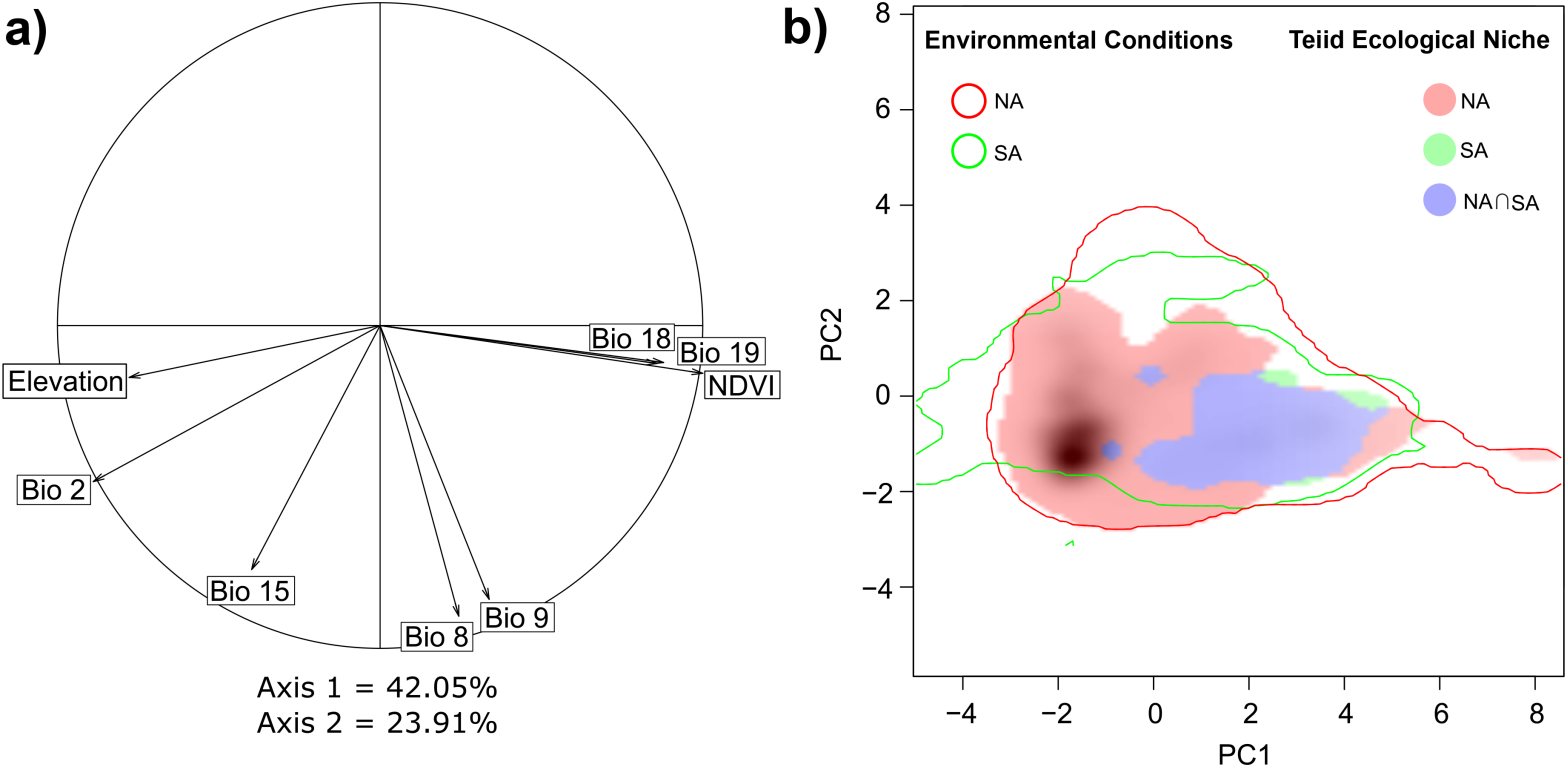
Correlation circle derived from the PCA of the environmental variables used to characterize the ecological niche of teiids (a) and niche overlap between North American teiids (*Aspidoscelis*, *Holcosus*, and *Pholidoscelis*) and their sister clade (*Ameiva*, *Cnemidophorus*, *Kentropyx*, and *Medopheos*) (b). In (a), the angles between predictors correspond to the correlation between them, and the projections of each vector on the axes are proportional to their contributions for each principal component. In (b), the axes depict the same principal components, and the lines represent the environmental conditions in North America (red) and South America (green). The blue stain corresponds to the niche overlap between both lineages, the red stain corresponds to the ecological niche exclusive to the northern lineage, and the green stain corresponds to the ecological niche exclusive to the southern lineage. The shading indicates the density of records of North American teiids.

**FIGURE 4:**
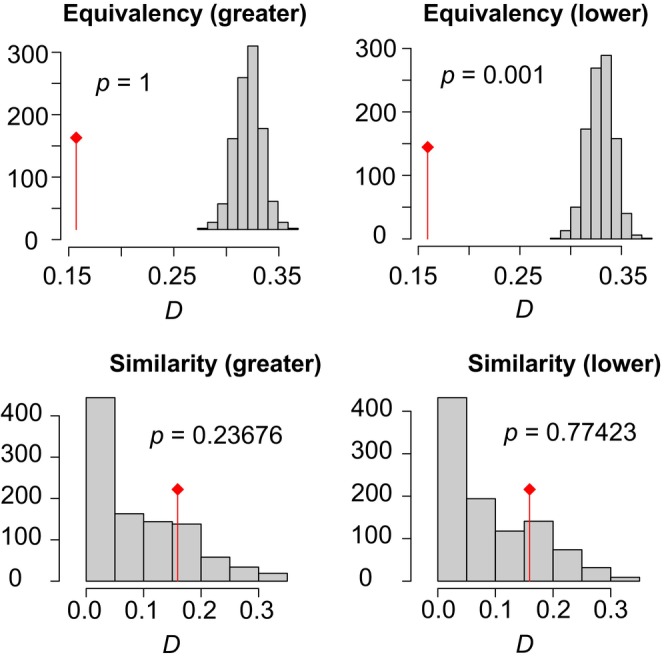
Histograms of simulated overlaps for equivalency and similarity tests. The text inside the parentheses indicates the alternative hypothesis tested: “higher” stands for “niche equivalency/similarity is higher than random,” and “lower” stands for “niche equivalency/similarity is lower than random.” The red line represents the observed Schoener's *D* value. The respective *p* values are shown in each graph.

The variables used to represent the ecological niche were elevation, NDVI, and the bioclimatic variables from WorldClim: Bio2, Bio8, Bio9, Bio15, Bio18, and Bio19 (Mean Diurnal Range, Mean Temperature of Wettest Quarter, Mean Temperature of Driest Quarter, Precipitation Seasonality, Precipitation of Warmest Quarter and Precipitation of Coldest Quarter). A correlation circle depicting all the environmental variables considered and the correlations between them is in Figure 3a. Plotting the ecological niches reveals that teiid's niche is broader in North America, and it encompasses most of the ecological conditions used by their sister clade, with only a small set of conditions used by the southern clade that are not colonized by the northern teiids (Figure 3b). Figure 3b also shows that the highest density of teiid records in North America comes from areas with different environmental conditions than the ones used by its sister lineage in South America. The observed Schoener's *D* value for niche overlap was 0.16. Niche equivalency tests showed that niches had lower overlap than random but niche similarity tests showed that niches were as similar to each other as random simulations (Figure 4). These results seem to go against our hypothesis of high niche overlap. Despite the low Schoener's D value, North American teiids had kept almost the entire ecological niche of their sister lineage—and putatively, their ancestors', which corroborates our last hypothesis, albeit in a way we did not anticipate.

## 4 Discussion

According to our first hypothesis, we found teiid speciation rates to be associated with the distribution range, with the North American range associated with higher rates in both GeoHiSSE runs. MiSSE analyses, on the other hand, seem to point to a different conclusion. Our first attempt to fit a MiSSE model was largely affected by a few short‐branched tips likely produced by hybridization. Our second attempt—after removing these species—resulted in a scenario where the main diversification rate increment happens much earlier than the migration itself. However, it is necessary to account for the fact that all species removed in the second MiSSE run have a North American distribution. This very likely caused diversification rates estimated for northern teiids to be underestimated. This bias could be partially bypassed if MiSSE allowed for clade‐specific sampling fractions. However, as MiSSE only supports a global sampling fraction (Vasconcelos, O'Meara, and Beaulieu 2022), which assumes random sampling through the tree, what effectively happened in our second MiSSE run was that the “weight” of missing northern species on diversification was shared with southern teiids. Because of this, we believe our MiSSE results should be interpreted cautiously. Curiously, removing these short‐branched tips did not decrease the importance of the distribution range, but rather gave a higher weight to the model including the distribution range and hidden states. Body size and body temperature were not associated with diversification. Contrary to our expectations, ecological niche similarity between North American teiids and their closest relatives was no different from random simulations, and niche equivalency was lower than random. However, we found that North American teiids occupy a broader ecological niche that encompasses almost completely and extends beyond the niche of its sister clade—which is close to our hypothesis of maintenance of ecological niche requirements. These results suggest that the North American lineage expanded the ancestral ecological niche upon colonizing new environments. Beyond that, we showed that the highest occurrence density of teiids in North America is registered in different environments from the utilized by its sister lineage in South America.

Although richness and body size differences between the two Teiidae subfamilies seem to follow a predicted pattern of an inverse relationship between body size and diversification rate (Hutchinson and Macarthur 1959), we did not find any relationship between them. Indeed, body size was shown previously to be unrelated to diversification in squamates (Feldman et al. 2016; Li and Wiens 2022). Our phylogeny is considerably comprehensive (over 60% of known teiid diversity), and we had a high coverage for this trait, so despite possible effects derived from incomplete taxon sampling, we think it is unlikely that body size influences diversification rates for this family. However, as body size is fairly simple to measure and readily available for many species (e.g., Meiri et al. 2018), we encourage other researchers to keep including it in their analyses. As for body temperatures, our results are not so reliable. This is because these data were only available for a small subset of species, and for many of them, minimum and maximum active body temperatures were the same, indicating that the measures were taken from only one individual (Meiri et al. 2018). Body temperatures in ectotherms are associated with habitat (Lanna et al. 2022), and since we observed that North American teiids managed to colonize different environmental conditions from their southern relatives, shifts in body temperatures are to be expected. Indeed, finding an association between body temperature and diversification should be more likely since physiological traits tend to be less phylogenetically conserved than body size (Blomberg, Garland Jr, and Ives 2003). Despite our results, many other physiology‐related traits, such as optimal and critical temperatures, may be even more relevant for ecology and diversification—albeit harder to obtain. These traits would likely play an essential role in such a habitat shift and, therefore, in the associated diversification. Another layer to this is that, unlike GeoHiSSE, ES‐sim does not account for incomplete taxon sampling on the phylogeny, which could greatly bias the results, especially if sampling is not random. Although our taxon sampling does not appear to be blatantly biased, the mere elongation of branch lengths derived from the exclusion of species could potentially diminish the diversification metric enough to affect the results.

The calculated Schoener's *D* statistic indicates low niche overlap (0.16). However, this is almost entirely due to the expansion of the ecological niche instead of divergence. Since Shoener's *D* is a symmetrical index, its value will be the same for the compared taxa, even though they differ greatly in breadth. Because of this, a low value of *D* may hide a scenario of almost complete overlap for the narrower niche, as is the case for these teiids. Broadening of the ecological niche and subsequent rapid speciation is an expected pattern following the colonization of a novel environment (Schluter 2000). However, the observed ecological differentiation was not accompanied by much phenotypical change, at least for the measured variables. Several hypotheses could be raised to explain this niche discrepancy, but we will highlight two. The first hypothesis is that the migrating teiids adapted to the new environment by evolving traits that relaxed their environmental requirements. Since North American teiids diversified so swiftly in arid areas markedly different from their ancestral southern range, we should expect to find an association between physiological traits—such as body temperature—and diversification. However, as discussed above, available data on body temperature may not be enough to detect this influence. Setting aside data limitations, yet another factor prevents us from completely discarding the effect of phenotype on teiid diversification. Since most northern teiids belong to the same clade, we cannot discard the possibility of a spurious correlation between distribution and diversification—the same pattern could arise due to the evolution of a trait in the ancestor of the northern radiation (Maddison and FitzJohn 2015). Indeed, this could be why the null model with hidden traits attained a moderate AIC weight. We fitted a MiSSE model to our tree to account for this possibility, but we do not think this was of much avail because of the previously discussed reasons. Alternatively, the variables we measured may not be relevant for teiid diversification. Investigations focusing on more phenotypical traits such as limb length, head shape, sprint speed, and others would help to clarify this point.

Another—more convoluted—possibility is that migrating teiids were preadapted to a broader range of environmental conditions than the ones they had access to in South America. One possibility is that the ancestors of northern teiids had comprehensive thermal tolerances and could easily live under higher temperatures than the ones they experienced in South America. This seems at odds with our finding that the environmental conditions of most areas occupied by teiids in North America are also available in South America, albeit unoccupied by these lizards. Why did the migrating teiids' closest relatives not colonize these habitats if they were preadapted to them? Perhaps these areas were inaccessible. We showed that most of the ecological niche expansion in the invaded continent was toward drier environments, where this lineage thrived the most. While dry areas are easily reachable in North America from the Isthmus of Panama, arid South American environments at low latitudes can only be found West of the Andes, a kind of barrier shown to be untraversable for other lizards (Ghaedi et al. 2021; Smissen et al. 2013). On the other hand, at intermediate latitudes, the Patagonian arid plains are too cold, and the low temperatures probably constrain the geographic distribution of the family (Jarnevich et al. 2018). Nevertheless, other teiids inhabit arid environments in trans‐Andean South America with relatively low diversity standards: only two species of *Callopistes* and three of *Dicrodon*. These genera, however, are more distantly related to the North American lineage, and other factors may play an important role—even more considering that the most diverse teiid genera can be grouped in a clade—the cnemidophorines—that does not include either. Another possibility, though, is that arid areas in trans‐Andean South America are not extensive enough to further the diversification of these lineages.

Another factor potentially affecting teiid diversification is the astonishing proportion of hybrid parthenogenetic species within Teiinae (Barley et al. 2022). Approximately 10% of the subfamily are exclusively parthenogenetic, and this proportion rises to 25% in *Aspidoscelis*. Unisexuality in squamates arises from hybridization (Fujita et al. 2020), which may generate novel species faster than cladogenesis—since no differentiation under reproductive isolation is required—which should inflate speciation rates. Moreover, parthenogenetic lizards were already suggested to have elevated speciation rates, albeit even higher extinction rates due to a lack of genetic variability and mutation accumulation, which explains why parthenogenetic species tend to be younger (Fujita et al. 2020; Moreira, Fonseca, and Rojas 2021). However, testing this hypothesis is not so straightforward since, in this case, diversification would not be driven by a binary trait (sexual vs. parthenogenetic) but rather by the propensity to hybridization, which is not so easily quantified. Speaking of hybridization, one criticism that may be raised against this work is that our analyses considered a bifurcating teiid tree—when, in fact, hybridizations make it to be reticulated. This is very much true, but it is also true that, currently, there are no algorithms able to run diversification analyses on reticulated trees, which makes the incorrect but valuable model of a bifurcating tree the only option for this kind of work.

Our results suggest that the teiid diversification burst after the colonization of North America was due to the great availability of suitable habitats. Faster diversification in newly colonized areas is congruent with the seizing of ecological opportunity and ecological release, and our results suggest that at least one of them took place at this point in teiid evolution. However, we do not feel confident about discarding an association between body temperature and diversification due to the low quality of the data. More insights on teiid diversification could come from assessing the effects of hybridization and other ecological variables related to physiology, morphology, and especially biotic interactions.

## Author Contributions

**Humberto Coelho Nappo:** data curation (lead), formal analysis (lead), investigation (lead), software (lead), visualization (lead), writing – original draft (lead). **Guarino Rinaldi Colli:** conceptualization (lead), funding acquisition (lead), resources (lead), supervision (lead), writing – review and editing (lead).

## Conflicts of Interest

The authors declare no conflicts of interest.

## Supporting information


Data S1.

## Data Availability

All data necessary to replicate our results is available in the Data S1.

## Appendix A

### A.1

Diversification analyses are sensitive to incomplete sampling, especially because taxa are not sampled randomly (Cusimano and Renner 2010; Höhna et al. 2011). Therefore, we followed a procedure to maximize the species included while avoiding using a low‐support topology. First, we updated the sequences used by Tonini et al. (2016) for all Teiidae and two Gymnophthalmidae species (*Cercosaura ocellata* and *Potamites ecpleopus*) with new sequences published in GenBank for the same genes: mitochondrial ribosomal RNA 12S and 16S, brain‐derived neurotrophic factor (BDNF), bone morphogenic protein 2 (BMP2), oocyte maturation factor mos (CMOS), cytochrome oxidase 1 (COI), cytochrome b (CYTB), NADH dehydrogenase subunits 1, 2, and 4 (ND1, ND2, and ND4), neurotrophin 3 (NT3), prolactin receptor (PRLR), G protein‐coupled receptor 149 (R35), and recombination activating genes 1 and 2 (RAG1 and RAG2). Tonini et al. (2016) also used amelogenin (AMEL) and phosducin (PDC) sequences, but we did not include these genes since there were no available AMEL sequences and only one PDC sequence published in GenBank for Teiidae.

From these sequences, we produced a multiple sequence alignment for each gene using the MAFFT algorithm in Geneious Prime 2022.2 (Biomatters 2022). After the automatic run, we checked and manually corrected all the alignments and excluded snippets with coverage lower than 80%. After this, we concatenated the alignments and subjected the full alignment to a constrained tree search in IQ‐TREE, 2.2.2.6 (Minh et al. 2020) with the MP‐EST topology from Tucker et al. (2016) as a constraint. Tucker et al. (2016) used a phylogenomic approach with many more loci and inferred highly congruent topologies with three different methods, albeit their analysis included only 56 species (32.4% of known diversity). Since we do not have the same data quality, we used their topology to constrain our search. Not all tips in the constraint tree were represented in our alignment since some did not have published sequences in GenBank. Because of this, we removed from the constraint tree tips that did not have counterparts in the alignment using the *drop. tip()* function of the “ape” package (Paradis and Schliep 2018). We used ModelFinder (Kalyaanamoorthy et al. 2017) to evaluate nucleotide substitution models separately for each gene and chose the best model according to the Bayesian Information Criterion (BIC). We assessed node support by running 1000 iterations of ultrafast bootstrap (Hoang et al. 2017).

Then, we inferred divergence times for the maximum likelihood tree with RelTime implementation in MEGA11 (Tamura, Stecher, and Kumar 2021; Tamura, Tao, and Kumar 2018) using default analysis settings for maximum likelihood branch lengths inference. We rooted the tree on the branch leading to *Cercosaura ocellata* and *Potamites ecpleopus* and calibrated it by assigning exponential prior distributions for the ages of two nodes based on fossil evidence. We defined the offset age for *Callopistes* as 20.1 Ma (Quadros, Chafrat, and Zaher 2018). For the origin of the clade *Pholidoscelis* + *Holcosus* + *Aspidoscelis*, we defined an offset age of 9.4 Ma (Scarpetta 2020). The final tree we used for the diversification analyses included 106 teiid species, representing around 60% of known diversity.
