# Supplementary material for: Colonization of North America Boosted the Diversification of Whiptail Lizards
Source: Ecol Evol. 2024 Oct 23;14(10):e70418. doi: 10.1002/ece3.70418 (PMC11496772; doi:10.1002/ece3.70418)
Supplement: Supplementary file 1 — Data S1. [file ECE3-14-e70418-s001.zip › ece370418-sup-0001-DataS1.docx]

**Supplementary File S1.** Body size, minimum, mean, and maximum body temperature for extant teiid species. The last four columns present references for the values shown. When the referenced article was not cited in the main text, we provide a link to it.

**Supplementary File S2.** Full Teiidae tree including 106 species.

**Supplementary File S3.** Area matrix codifying the distribution range of teiid species for GeoHiSSE analysis with the full Teiidae tree. The distribution range categories are South America (1), North America (2), and both (0).

**Supplementary File S4.** Area matrix codifying the distribution range of teiid species for GeoHiSSE analysis without overly short branches (pruned tree). The distribution range categories are South America (1), North America (2), and both (0).

**Supplementary File S5.** R code used to run the GeoHiSSE, ES-sim, and niche overlap analyses. We could not include the code for MiSSE analyses due to computational limitations. However, we used the same code provided by Vasconcelos et al. (2022) with the sampling fraction adjustments detailed in the methods section.
