# Supplementary material for: Colonization of North America Boosted the Diversification of Whiptail Lizards
Source: Ecol Evol. 2024 Oct 23;14(10):e70418. doi: 10.1002/ece3.70418 (PMC11496772; doi:10.1002/ece3.70418)

Teiidae Diversification and Niche Overlap

Humberto C. Nappo and Guarino R. Colli

Table of contents

1 Load Packages 2

2 State-Dependent Diversification 3
2.1 GeoHiSSE with full tree . . . 3
2.1.1 Plot GeoHiSSE Phylogram . . . 7
2.2 GeoHiSSE with pruned tree . . . 8
2.2.1 Plot GeoHiSSE Phylogram . . . 13
2.3 ES-SIM . . . 14

3 Niche Overlap 17
3.1 Download registers from GBIF . . . 17
3.2 Plot Registers . . . 20
3.3 Clean Coordinates . . . 22
3.4 Download Environmental Variables . . . 29
3.5 Generate Pseudoabsences . . . 32
3.6 Calculate Variance Inflation Factors (VIFs) . . . 32
3.7 Extract Environmental Variable Data for Each Coordinate . . . 33
3.8 Ordinate Environmental Conditions . . . 34
3.9 Calculate Ecological Niches in Native and Invaded Ranges . . . 35
3.10 Niche Equivalency and Similarity Tests . . . 36
3.11 Niche Equivalency/Similarity Histograms . . . 38
3.12 Niche Overlap Graph . . . 39

1 Load Packages

```
#install.packages("ade4", dep = T)
#install.packages("ape", dep = T)
#install.packages("CoordinateCleaner", dep = T)
#install.packages("countrycode", dep = T)
#install.packages("dispRity", dep = T)
#install.packages("ecospat", dep = T)
#install.packages("geodata", dep = T)
#install.packages("hisse", dep = T)
#install.packages("MODIStsp", dep = T)
#install.packages("nlme", dep = T)
#install.packages("parallel", dep = T)
#install.packages("readxl", dep = T)
#install.packages("rgbif", dep = T)
#install.packages("Rphylopars", dep = T)
#install.packages("rnaturalearth", dep = T)
#install.packages("sf", dep = T)
#install.packages("strap", dep  =T)
#install.packages("terra", dep = T)
#install.packages("tidyterra", dep = T)
#install.packages("tidyverse", dep = T)
#install.packages("usdm", dep = T)

library(ade4)
library(ape)
library(CoordinateCleaner)
library(countrycode)
library(dispRity)
library(ecospat)
library(geodata)
library(hisse)
library(MODIStsp)
library(nlme)
library(parallel)
library(readxl)
library(rgbif)
library(Rphylopars)
library(rnaturalearth)
library(sf)
library(strap)
```

```r
library(terra)
library(tidyterra)
library(tidyverse)
library(usdm)
```

2 State-Dependent Diversification

2.1 GeoHiSSE with full tree

Import Teiidae phylogeny and area matrix with the classification of the distribution range of teiids. Distribution ranges are classified as follows:

(1) South America: mainland South America and islands outside the Caribbean Sea

(2) North America: Central America, North America, and Caribbean islands

(0) both: mainland South America AND (Central/North America OR Caribbean islands)

```r
# Import Teiidae phylogeny
Teiidae_tree <- read.tree("Teiidae_tree.tre")

# Import Area Matrix
area_matrix <- read.table("Area_Matrix.txt")
```

Build GeoHiSSE models. The `f` argument of `GeoHiSSE()` function corresponds to the sampled fraction for South America, North America, and both, in this order.

```r
# Model 1: Range-independent, no hidden states
trans.rate <- TransMatMakerGeoHiSSE(hidden.traits = 0)
trans.rate.mod <- ParEqual(trans.rate, c(1,2))
mod1 <- GeoHiSSE(phy = Teiidae_tree,
               data = area_matrix,
               f = c(0.5270,0.6556,0.8),
               turnover = c(1,1,0),
               eps = c(1,1),
               hidden.states = FALSE,
               trans.rate = trans.rate.mod,
               turnover.upper = 100,
               trans.upper = 10)
```

```
Initializing...
```

```
Finished. Beginning simulated annealing...
Finished. Refining using subplex routine...
Finished. Summarizing results...
```

```
# Model 2: Range-dependent, no hidden states
trans.rate <- TransMatMakerGeoHiSSE(hidden.traits = 0)
trans.rate.mod <- ParEqual(trans.rate, c(1,2))
mod2 <- GeoHiSSE(phy = Teiidae_tree,
                 data = area_matrix,
                 f = c(0.5270,0.6556,0.8),
                 turnover = c(1,2,3),
                 eps = c(1,1),
                 hidden.states = FALSE,
                 trans.rate = trans.rate.mod,
                 turnover.upper = 100,
                 trans.upper=10)
```

```
Initializing...
Finished. Beginning simulated annealing...
Finished. Refining using subplex routine...
Finished. Summarizing results...
```

```
# Model 3: Range-independent + hidden states
trans.rate <- TransMatMakerGeoHiSSE(hidden.traits = 1,
                                   make.null = TRUE)
trans.rate.mod <- ParEqual(trans.rate, c(1,2))

mod3 <- GeoHiSSE(phy = Teiidae_tree,
                       data = area_matrix,
                       f = c(0.5270,0.6556,0.8),
                       turnover = c(1,1,0,2,2,0),
                       eps = c(1,1,1,1),
                       hidden.states = TRUE,
                       trans.rate = trans.rate.mod,
                       turnover.upper = 100,
                       trans.upper = 10)
```

```
Initializing...
Finished. Beginning simulated annealing...
Finished. Refining using subplex routine...
Finished. Summarizing results...
```

```r
# Model 4: Range-dependent + hidden states
trans.rate <- TransMatMakerGeoHiSSE(hidden.traits = 1)
trans.rate.mod <- ParEqual(trans.rate,c(1,2))

mod4 <- GeoHiSSE(phy = Teiidae_tree,
                           data = area_matrix,
                           f = c(0.5270,0.6556,0.8),
                           turnover = c(1,2,3,4,5,6),
                           eps = c(1,1,1,1),
                           hidden.states = TRUE,
                           trans.rate = trans.rate.mod,
                           turnover.upper = 100,
                           trans.upper = 10)
```

```
Initializing...
Finished. Beginning simulated annealing...
Finished. Refining using subplex routine...
Finished. Summarizing results...
```

Calculate the weighted average of all models.

```r
# Get AIC weights
GetAICWeights(list(mod1, mod2, mod3, mod4),
              criterion="AIC")
```

```
[1] 2.456636e-05 6.949658e-01 3.050069e-01 2.766519e-06
```

```r
recon_mod1 <- MarginReconGeoSSE(phy = mod1$phy,
                                data = mod1$data,
                                f = mod1$f,
                                pars = mod1$solution,
                                hidden.states = 1,
                                root.type = mod1$root.type,
                                root.p = mod1$root.p,
                                AIC = mod1$AIC,
                                n.cores = 1)
```

```
Calculating marginal probabilities for 105 internal nodes...
Done.
```

```r
recon_mod2 <- MarginReconGeoSSE(phy = mod2$phy,
                                data = mod2$data,
                                f = mod2$f,
                                pars = mod2$solution,
                                hidden.states = 1,
                                root.type = mod2$root.type,
                                root.p = mod2$root.p,
                                AIC = mod2$AIC,
                                n.cores = 1)
```

```
Calculating marginal probabilities for 105 internal nodes...
Done.
```

```r
recon_mod3 <- MarginReconGeoSSE(phy = mod3$phy,
                                  data = mod3$data,
                                  f = mod3$f,
                                  pars = mod3$solution,
                                  hidden.states = 2,
                                  root.type = mod3$root.type,
                                  root.p = mod3$root.p,
                                  AIC = mod3$AIC,
                                  n.cores = 1)
```

```
Calculating marginal probabilities for 105 internal nodes...
Finished. Calculating marginal probabilities for 106 tips...
Done.
```

```r
recon_mod4 <- MarginReconGeoSSE(phy = mod4$phy,
                                  data = mod4$data,
                                  f = mod4$f,
                                  pars = mod4$solution,
                                  hidden.states = 2,
                                  root.type = mod4$root.type,
                                  root.p = mod4$root.p,
                                  AIC = mod4$AIC,
                                  n.cores = 1)
```

```
Calculating marginal probabilities for 105 internal nodes...
Finished. Calculating marginal probabilities for 106 tips...
Done.
```

```r
recon_models <- list(recon_mod1,
                     recon_mod2,
                     recon_mod3,
                     recon_mod4)

model_ave_rates <- GetModelAveRates(x = recon_models, type = "tips")
```

2.1.1 Plot GeoHiSSE Phylogram

Plot a phylogram depicting diversification rates and distribution range classification. Outline colors correspond to diversification rates following the inset legend. Branch colors correspond to the distribution range: white for South America, black for North America, and yellow for both.

```r
# Plot
plot.geohisse.states(x = recon_models,
                     rate.param = "speciation",
                     type = "fan",
                     show.tip.label = FALSE,
                     legend = TRUE,
                     state.colors = c("white","black","gray"),
                     legend.kernel = "traditional",
                     edge.width = 12,
                     width.factor = 0.4)
```

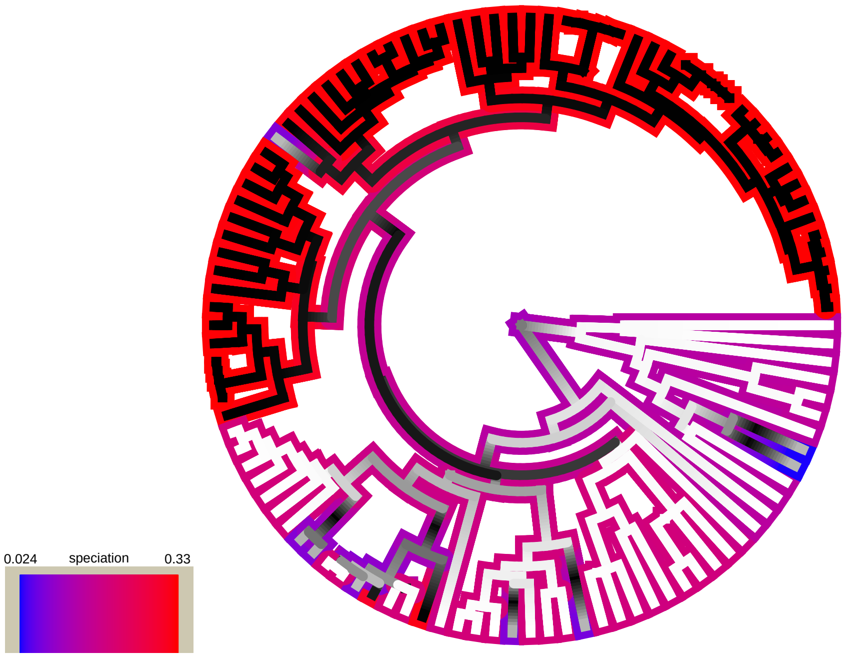


```
$rate.tree
Object of class "contMap" containing:

(1) A phylogenetic tree with 106 tips and 105 internal nodes.

(2) A mapped continuous trait on the range (0.024047, 0.334837).


$state.tree
Object of class "contMap" containing:

(1) A phylogenetic tree with 106 tips and 105 internal nodes.

(2) A mapped continuous trait on the range (0, 2.002).
```

2.2 GeoHiSSE with pruned tree

In addition to running GeoHiSSE with our full tree, we also ran it removing excessively short branches.

```r
# Pruned tree
pruned_tree <- ape::drop.tip(Teiidae_tree, tip = c("Pholidoscelis_corvinus",
                                                    "Pholidoscelis_corax",
                                                    "Aspidoscelis_marmoratus",
                                                    "Aspidoscelis_tigris",
                                                    "Aspidoscelis_neotesselatus",
                                                    "Aspidoscelis_sonorae",
                                                    "Aspidoscelis_uniparens",
                                                    "Aspidoscelis_opatae"))
# Pruned area matrix
pruned_matrix <- read.table("Pruned_Area_Matrix.txt")
```

Build GeoHiSSE models. The `f` argument of `GeoHiSSE()` function corresponds to the sampled fraction for South America, North America, and both, in this order.

```r
# Model 1: Range-independent, no hidden states
trans.rate <- TransMatMakerGeoHiSSE(hidden.traits = 0)
trans.rate.mod <- ParEqual(trans.rate, c(1,2))
mod1 <- GeoHiSSE(phy = pruned_tree,
               data = pruned_matrix,
               f = c(0.5270,0.5667,0.8),
               turnover = c(1,1,0),
               eps = c(1,1),
               hidden.states = FALSE,
               trans.rate = trans.rate.mod,
               turnover.upper = 100,
               trans.upper = 10)
```

```
Initializing...
Finished. Beginning simulated annealing...
Finished. Refining using subplex routine...
Finished. Summarizing results...
```

```r
# Model 2: Range-dependent, no hidden states
trans.rate <- TransMatMakerGeoHiSSE(hidden.traits = 0)
trans.rate.mod <- ParEqual(trans.rate, c(1,2))
mod2 <- GeoHiSSE(phy = pruned_tree,
                 data = pruned_matrix,
                 f = c(0.5270,0.5667,0.8),
                 turnover = c(1,2,3),
                 eps = c(1,1),
```

```
                   hidden.states = FALSE,
                   trans.rate = trans.rate.mod,
                   turnover.upper = 100,
                   trans.upper=10)
```

```
Initializing...
Finished. Beginning simulated annealing...
Finished. Refining using subplex routine...
Finished. Summarizing results...
```

```
# Model 3: Range-independent + hidden states
trans.rate <- TransMatMakerGeoHiSSE(hidden.traits = 1,
                                    make.null = TRUE)
trans.rate.mod <- ParEqual(trans.rate, c(1,2))

mod3 <- GeoHiSSE(phy = pruned_tree,
                       data = pruned_matrix,
                       f = c(0.5270,0.5667,0.8),
                       turnover = c(1,1,0,2,2,0),
                       eps = c(1,1,1,1),
                       hidden.states = TRUE,
                       trans.rate = trans.rate.mod,
                       turnover.upper = 100,
                       trans.upper = 10)
```

```
Initializing...
Finished. Beginning simulated annealing...
Finished. Refining using subplex routine...
Finished. Summarizing results...
```

```
# Model 4: Range-dependent + hidden states
trans.rate <- TransMatMakerGeoHiSSE(hidden.traits = 1)
trans.rate.mod <- ParEqual(trans.rate,c(1,2))

mod4 <- GeoHiSSE(phy = pruned_tree,
                           data = pruned_matrix,
                           f = c(0.5270,0.5667,0.8),
                           turnover = c(1,2,3,4,5,6),
                           eps = c(1,1,1,1),
```

```
                            hidden.states = TRUE,
                            trans.rate = trans.rate.mod,
                            turnover.upper = 100,
                            trans.upper = 10)
```

```
Initializing...
Finished. Beginning simulated annealing...
Finished. Refining using subplex routine...
Finished. Summarizing results...
```

Calculate the weighted average of all models.

```
# Get AIC weights
GetAICWeights(list(mod1, mod2, mod3, mod4),
              criterion="AIC")
```

```
[1] 5.521318e-06 5.707348e-02 2.155789e-02 9.213631e-01
```

```
recon_mod1 <- MarginReconGeoSSE(phy = mod1$phy,
                                data = mod1$data,
                                f = mod1$f,
                                pars = mod1$solution,
                                hidden.states = 1,
                                root.type = mod1$root.type,
                                root.p = mod1$root.p,
                                AIC = mod1$AIC,
                                n.cores = 1)
```

```
Calculating marginal probabilities for 97 internal nodes...
Done.
```

```
recon_mod2 <- MarginReconGeoSSE(phy = mod2$phy,
                              data = mod2$data,
                              f = mod2$f,
                              pars = mod2$solution,
                              hidden.states = 1,
                              root.type = mod2$root.type,
                              root.p = mod2$root.p,
```

```
                                AIC = mod2$AIC,
                                n.cores = 1)
```

```
Calculating marginal probabilities for 97 internal nodes...
Done.
```

```
recon_mod3 <- MarginReconGeoSSE(phy = mod3$phy,
                                data = mod3$data,
                                f = mod3$f,
                                pars = mod3$solution,
                                hidden.states = 2,
                                root.type = mod3$root.type,
                                root.p = mod3$root.p,
                                AIC = mod3$AIC,
                                n.cores = 1)
```

```
Calculating marginal probabilities for 97 internal nodes...
Finished. Calculating marginal probabilities for 98 tips...
Done.
```

```
recon_mod4 <- MarginReconGeoSSE(phy = mod4$phy,
                                data = mod4$data,
                                f = mod4$f,
                                pars = mod4$solution,
                                hidden.states = 2,
                                root.type = mod4$root.type,
                                root.p = mod4$root.p,
                                AIC = mod4$AIC,
                                n.cores = 1)
```

```
Calculating marginal probabilities for 97 internal nodes...
Finished. Calculating marginal probabilities for 98 tips...
Done.
```

```
recon_models <- list(recon_mod1,
                     recon_mod2,
                     recon_mod3,
                     recon_mod4)
```

```r
model_ave_rates <- GetModelAveRates(x = recon_models, type = "tips")
```

2.2.1 Plot GeoHiSSE Phylogram

Plot a phylogram depicting diversification rates and distribution range classification. Outline colors correspond to diversification rates following the inset legend. Branch colors correspond to the distribution range: white for South America, black for North America, and yellow for both.

```r
# Plot
plot.geohisse.states(x = recon_models,
                     rate.param = "speciation",
                     type = "fan",
                     show.tip.label = FALSE,
                     legend = TRUE,
                     state.colors = c("white","black","gray"),
                     legend.kernel = "traditional",
                     edge.width = 12,
                     width.factor = 0.4)
```

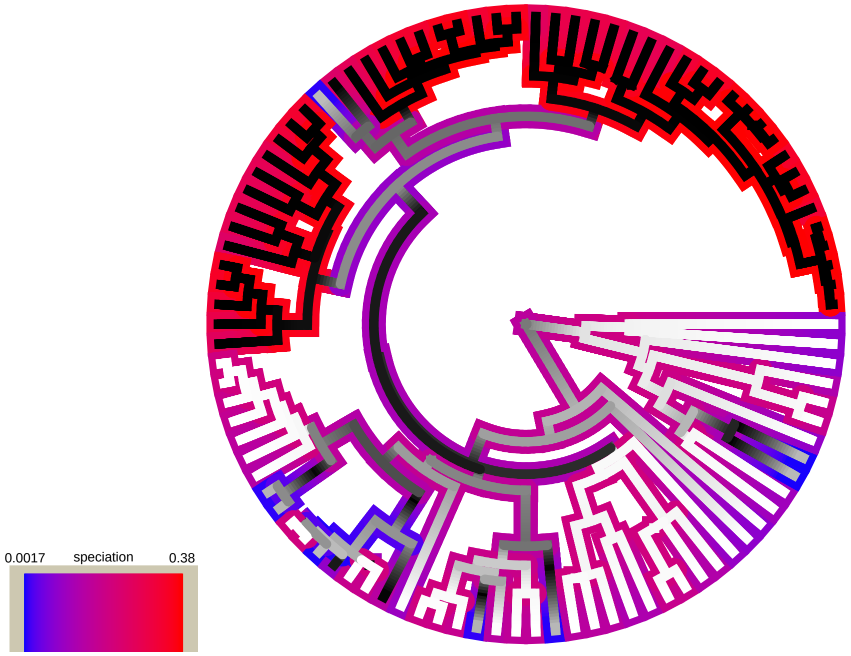


```
$rate.tree
```

```
Object of class "contMap" containing:

(1) A phylogenetic tree with 98 tips and 97 internal nodes.

(2) A mapped continuous trait on the range (0.001704, 0.382111).


$state.tree
Object of class "contMap" containing:

(1) A phylogenetic tree with 98 tips and 97 internal nodes.

(2) A mapped continuous trait on the range (0, 2.002).
```

2.3 ES-SIM

Import body size and body temperature data

```
# Load trait data
traits <- readxl::read_xlsx("Teiidae_traits.xlsx")[,c(1:5)]
```

Impute missing data

```
# Impute missing data for SVL and temperatures
imputation <- Rphylopars::phylopars(traits, Teiidae_tree)


# Define predictors (imputed SVL and temperatures)
imp_max_SVL <- imputation[["anc_recon"]][c(1:106), "max_SVL"]
imp_min_Tb <- imputation[["anc_recon"]][c(1:106), "mean_min_Tb"]
imp_mean_Tb <- imputation[["anc_recon"]][c(1:106), "mean_Tb"]
imp_max_Tb <- imputation[["anc_recon"]][c(1:106), "mean_max_Tb"]

imputed_predictors <- cbind.data.frame(imp_max_SVL,
                                       imp_min_Tb,
                                       imp_mean_Tb,
                                       imp_max_Tb) %>%
  tibble::rownames_to_column("species")
```

Run inverse equal-pslits with simulations tests (Es-sim). The `essim.R` file may be downloaded from Harvey & Rabosky (2018).

```r
# Load function for testing association between trait and diversification
source("essim.R")
```

```
Carregando pacotes exigidos: mvtnorm

Carregando pacotes exigidos: phangorn
```

```r
# Test with imputed maximum SVL
essim_Imp_SVL <- essim(Teiidae_tree,
                       imputation[["anc_recon"]][c(1:106), "max_SVL"])

# Test with imputed mean minimum body temperature
essim_Imp_minTb <- essim(Teiidae_tree,
                         imputation[["anc_recon"]][c(1:106), "mean_min_Tb"])

# Test with imputed mean body temperature
essim_Imp_meanTb <- essim(Teiidae_tree,
                          imputation[["anc_recon"]][c(1:106), "mean_Tb"])

# Test with imputed mean maximum body temperature
essim_Imp_maxTb <- essim(Teiidae_tree,
                         imputation[["anc_recon"]][c(1:106), "mean_max_Tb"])


# Test with available maximum SVL data (without imputed data)
av_max_SVL <- traits[,c("species","max_SVL")] %>%
  filter(., complete.cases(.$max_SVL)) %>%
  deframe(.)

essim_SVL <- essim(drop.tip(Teiidae_tree,
                            sort(Teiidae_tree$tip.label)[is.na(traits$max_SVL) == T]),
                   av_max_SVL)

# Test with available minimum body temperature data (without imputed data)
av_min_Tb <- traits[,c("species","mean_min_Tb")] %>%
  filter(., complete.cases(.$mean_min_Tb)) %>%
  deframe(.)

essim_minTb <- essim(drop.tip(Teiidae_tree,
                              sort(Teiidae_tree$tip.label)[is.na(traits$mean_min_Tb) == T]
                     av_min_Tb)
```

```r
# Test with available mean body temperature data (without imputed data)
av_mean_Tb <- traits[,c("species","mean_Tb")] %>%
  filter(., complete.cases(.$mean_Tb)) %>%
  deframe(.)

essim_meanTb <- essim(drop.tip(Teiidae_tree,
                               sort(Teiidae_tree$tip.label)[is.na(traits$mean_Tb) == T]),
                      av_mean_Tb)

# Test with available maximum body temperature data (without imputed data)
av_max_Tb <- traits[,c("species","mean_max_Tb")] %>%
  filter(., complete.cases(.$mean_max_Tb)) %>%
  deframe(.)

essim_maxTb <- essim(drop.tip(Teiidae_tree,
                              sort(Teiidae_tree$tip.label)[is.na(traits$mean_max_Tb) == T]
                     av_max_Tb)


# Make a table with all Es-sim results
(essim_table <- rbind(essim_Imp_SVL,
                     essim_Imp_minTb,
                     essim_Imp_meanTb,
                     essim_Imp_maxTb,
                     essim_SVL,
                     essim_minTb,
                     essim_meanTb,
                     essim_maxTb))
```

```
                        rho   P Value
essim_Imp_SVL    -0.4075660 0.1838162
essim_Imp_minTb   0.2050815 0.5274725
essim_Imp_meanTb  0.2688442 0.3816184
essim_Imp_maxTb   0.3280757 0.2837163
essim_SVL        -0.4040589 0.1738262
essim_minTb       0.3260446 0.3476523
essim_meanTb      0.4072709 0.2497502
essim_maxTb       0.4133281 0.2557443
```

3 Niche Overlap

Assess ecological niche overlap between North American teiines: *Aspidoscelis*, *Holcosus*, and *Pholidoscelis* (hereafter, North American teiines) and their closest South American relatives: *Ameiva*, *Cnemidophorus*, *Kentropyx*, and *Medopheos* (hereafter, South American teiines).

3.1 Download registers from GBIF

Download Teiidae registers from Global Biodiversity Information Facility (GBIF). The `rgbif::occ_download()` function requires a registration in GBIF and demands the user to provide an email, username and password to request the download.

```
# Define genera that will be used for niche overlap analyses
SA_teiines <- c("Ameiva", "Cnemidophorus", "Kentropyx", "Medopheos")
NA_teiines <- c("Aspidoscelis", "Holcosus", "Pholidoscelis")

# Get genera keys from GBIF
SA_teiines_key <- vector()
NA_teiines_key <- vector()

for (i in 1:length(SA_teiines)) {

  # South American teiines
  SA_teiines_key[i] <- rgbif::name_backbone(name = SA_teiines[i],
                                            genus = SA_teiines[i])$genusKey

  # North American teiines
  ifelse(i <= length(NA_teiines),
         NA_teiines_key[i] <- rgbif::name_backbone(name = NA_teiines[i],
                                                   genus = NA_teiines[i])$genusKey,
         NA)
}


# Download GBIF data
#rgbif::occ_download(pred_or(pred("taxonKey", SA_teiines_key[1]),
#                            pred("taxonKey", SA_teiines_key[2]),
#                            pred("taxonKey", SA_teiines_key[3]),
#                            pred("taxonKey", SA_teiines_key[4])),
#                    pred("hasCoordinate", T),
#                    pred("hasGeospatialIssue", F),
```

```
#                     pred("occurrenceStatus", "PRESENT"),
#                     type = "and",
#                     user = "user",
#                     email = "email",
#                     pwd = "password",
#                     format = "SIMPLE_CSV")

#rgbif::occ_download(pred_or(pred("taxonKey", NA_teiines_key[1]),
#                             pred("taxonKey", NA_teiines_key[2]),
#                             pred("taxonKey", NA_teiines_key[3])),
#                     pred("hasCoordinate", T),
#                     pred("hasGeospatialIssue", F),
#                     pred("occurrenceStatus", "PRESENT"),
#                     type = "and",
#                     user = "user",
#                     email = "email",
#                     pwd = "password",
#                     format = "SIMPLE_CSV")


# Load South American teiines GBIF data
SA_teiines_occ <- read.table("SA_teiines_occ_GBIF.csv",
                             header = T,
                             sep = "\t",
                             fill = T,
                             quote = "") %>%

  # Remove unnecessary columns
  dplyr::select(.,
                species,
                decimalLongitude,
                decimalLatitude,
                occurrenceStatus,
                individualCount,
                genus,
                taxonRank,
                countryCode,
                coordinateUncertaintyInMeters,
                year,
                basisOfRecord,
                gbifID,
```

```
                institutionCode) %>%

  # Remove rows without coordinates
  filter(!is.na(decimalLongitude)) %>%
  filter(!is.na(decimalLatitude)) %>%

  # Remove registers that are not associated with preserved specimens
  filter(basisOfRecord == "PRESERVED_SPECIMEN") %>%

  # Remove registers of 0 individuals
  filter(individualCount > 0 | is.na(individualCount)) %>%

  # Remove registers of unidentified species
  filter(!species == "") %>%

  # Remove registers of unidentified institutions
  filter(!institutionCode == "") %>%

  # Remove old registers
  filter(year > 2000)


# Load North American teiines GBIF data
NA_teiines_occ <- read.table("NA_teiines_occ_GBIF.csv",
                             header = T,
                             sep = "\t",
                             fill = T,
                             quote = "") %>%

  # Remove unnecessary columns
  dplyr::select(.,
         species,
         decimalLongitude,
         decimalLatitude,
         occurrenceStatus,
         individualCount,
         genus,
         taxonRank,
         countryCode,
         coordinateUncertaintyInMeters,
         year,
```

```
         basisOfRecord,
         gbifID,
         institutionCode) %>%

  # Remove rows without coordinates
  filter(!is.na(decimalLongitude)) %>%
  filter(!is.na(decimalLatitude)) %>%

  # Remove registers that are not associated with preserved specimens
  filter(basisOfRecord == "PRESERVED_SPECIMEN") %>%

  # Remove registers of 0 individuals
  filter(individualCount > 0 | is.na(individualCount)) %>%

  # Remove registers of unidentified species
  filter(!species == "") %>%

  # Remove registers of unidentified institutions
  filter(!institutionCode == "") %>%

  # Remove old registers
  filter(year > 2000)
```

3.2 Plot Registers

```
# Plot registers
wm <- borders("world", colour="gray50", fill="gray50")

ggplot() +
  coord_fixed() +
  wm +
  geom_point(data = SA_teiines_occ,
             aes(x = decimalLongitude, y = decimalLatitude),
             colour = "darkred", size = 0.5)+
  theme_bw()
```

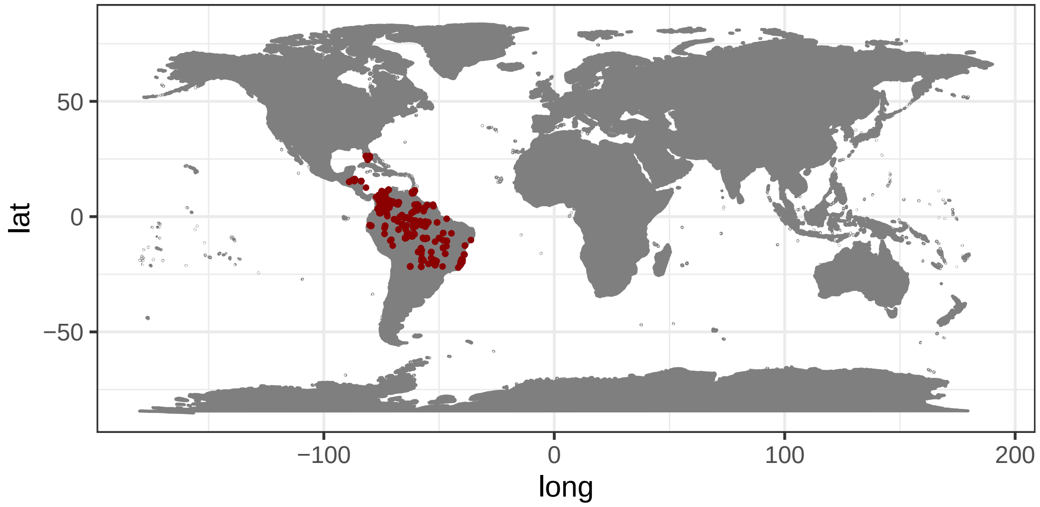


```r
ggplot() +
  coord_fixed() +
  wm +
  geom_point(data = NA_teiines_occ,
             aes(x = decimalLongitude, y = decimalLatitude),
             colour = "darkred", size = 0.5)+
  theme_bw()
```

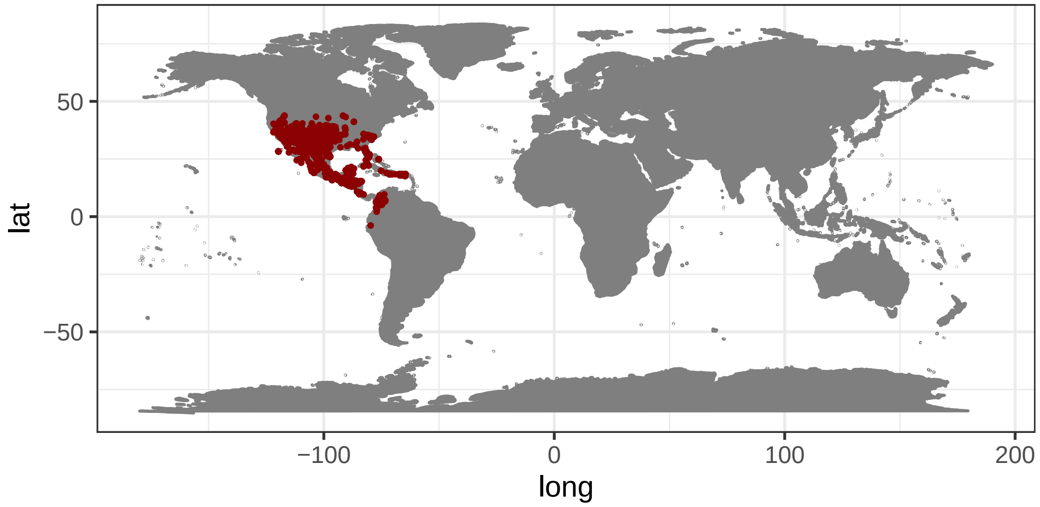

3.3 Clean Coordinates

Remove registers with common spatial issues.

```
# Convert country codes from ISO2c to ISO3c
SA_teiines_occ$countryCode <-  countrycode(SA_teiines_occ$countryCode,
                                           origin =  'iso2c',
                                           destination = 'iso3c')

NA_teiines_occ$countryCode <-  countrycode(NA_teiines_occ$countryCode,
                                           origin =  'iso2c',
                                           destination = 'iso3c')

# Flag problematic records
flags_SA <- clean_coordinates(x = SA_teiines_occ,
                              lon = "decimalLongitude",
                              lat = "decimalLatitude",
                              countries = "countryCode",
                              species = "species",
                              seas_ref = rnaturalearth::ne_download(scale = 10,
                                                                     type = 'land',
```

```
                                                                              category = 'physical')
                                seas_scale = 110,
                                tests = c("capitals",
                                          "centroids",
                                          "equal",
                                          "gbif",
                                          "institutions",
                                          "zeros",
                                          "countries",
                                          "urban",
                                          "duplicates",
                                          "seas",
                                          "outliers"))
```

```
Testing coordinate validity

Flagged 0 records.

Testing equal lat/lon

Flagged 0 records.

Testing zero coordinates

Flagged 0 records.

Testing country capitals

Flagged 1 records.

Testing country centroids

Flagged 0 records.

Testing sea coordinates
```

```
Reading layer `ne_10m_land' from data source
  `C:\Users\Usuario\AppData\Local\Temp\RtmpC49mmo\ne_10m_land.shp'
  using driver `ESRI Shapefile'
Simple feature collection with 11 features and 3 fields
Geometry type: MULTIPOLYGON
Dimension:     XY
Bounding box:  xmin: -180 ymin: -90 xmax: 180 ymax: 83.6341
Geodetic CRS:  WGS 84

Flagged 33 records.

Testing urban areas

Downloading urban areas via rnaturalearth

Reading layer `ne_50m_urban_areas' from data source
  `C:\Users\Usuario\AppData\Local\Temp\RtmpC49mmo\ne_50m_urban_areas.shp'
  using driver `ESRI Shapefile'
Simple feature collection with 2143 features and 4 fields
Geometry type: POLYGON
Dimension:     XY
Bounding box:  xmin: -157.984 ymin: -46.26844 xmax: 174.97 ymax: 69.35127
Geodetic CRS:  WGS 84

Flagged 31 records.

Testing country identity

Flagged 69 records.

Testing geographic outliers

Flagged 5 records.

Testing GBIF headquarters, flagging records around Copenhagen

Flagged 0 records.

Testing biodiversity institutions
```

```
Flagged 0 records.

Testing duplicates

Flagged 736 records.

Flagged 777 of 1266 records, EQ = 0.61.
```

```r
summary(flags_SA)
```

```
    .val      .equ      .zer      .cap      .cen      .sea      .urb      .con 
       0         0         0         1         0        33        31        69 
    .otl      .gbf     .inst      .dpl  .summary 
       5         0         0       736       777 
```

```r
plot(flags_SA, lon = "decimalLongitude", lat = "decimalLatitude")
```

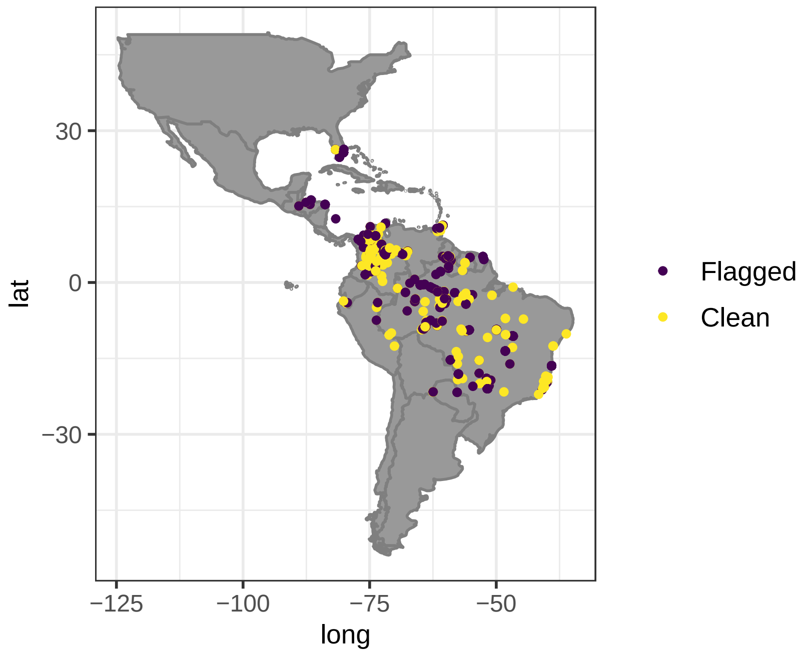

```r
flags_NA <- clean_coordinates(x = NA_teiines_occ,
                              lon = "decimalLongitude",
                              lat = "decimalLatitude",
                              countries = "countryCode",
                              species = "species",
                              seas_ref = rnaturalearth::ne_download(scale = 10,
                                                                     type = 'land',
                                                                     category = 'physical')
                              seas_scale = 110,
                              tests = c("capitals",
                                        "centroids",
                                        "equal",
                                        "gbif",
                                        "institutions",
                                        "zeros",
                                        "countries",
                                        "urban",
                                        "duplicates",
                                        "seas",
                                        "outliers"))
```

```
Testing coordinate validity

Flagged 0 records.

Testing equal lat/lon

Flagged 0 records.

Testing zero coordinates

Flagged 0 records.

Testing country capitals

Flagged 14 records.

Testing country centroids
```

```
Flagged 0 records.

Testing sea coordinates

Reading layer `ne_10m_land' from data source
  `C:\Users\Usuario\AppData\Local\Temp\RtmpC49mmo\ne_10m_land.shp'
  using driver `ESRI Shapefile'
Simple feature collection with 11 features and 3 fields
Geometry type: MULTIPOLYGON
Dimension:     XY
Bounding box:  xmin: -180 ymin: -90 xmax: 180 ymax: 83.6341
Geodetic CRS:  WGS 84

Flagged 125 records.

Testing urban areas

Downloading urban areas via rnaturalearth

Reading layer `ne_50m_urban_areas' from data source
  `C:\Users\Usuario\AppData\Local\Temp\RtmpC49mmo\ne_50m_urban_areas.shp'
  using driver `ESRI Shapefile'
Simple feature collection with 2143 features and 4 fields
Geometry type: POLYGON
Dimension:     XY
Bounding box:  xmin: -157.984 ymin: -46.26844 xmax: 174.97 ymax: 69.35127
Geodetic CRS:  WGS 84

Flagged 135 records.

Testing country identity

Flagged 134 records.

Testing geographic outliers

Warning in cc_outl(otl_test, lon = lon, lat = lat, species = species, method =
outliers_method, : Species with fewer than 7 unique records will not be tested.
```

```
Flagged 273 records.

Testing GBIF headquarters, flagging records around Copenhagen

Flagged 0 records.

Testing biodiversity institutions

Flagged 2 records.

Testing duplicates

Flagged 3803 records.

Flagged 4131 of 7326 records, EQ = 0.56.
```

```r
summary(flags_NA)
```

```
    .val      .equ      .zer      .cap      .cen      .sea      .urb      .con
       0         0         0        14         0       125       135       134
    .otl      .gbf     .inst      .dpl  .summary
     273         0         2      3803      4131
```

```r
plot(flags_NA, lon = "decimalLongitude", lat = "decimalLatitude")
```

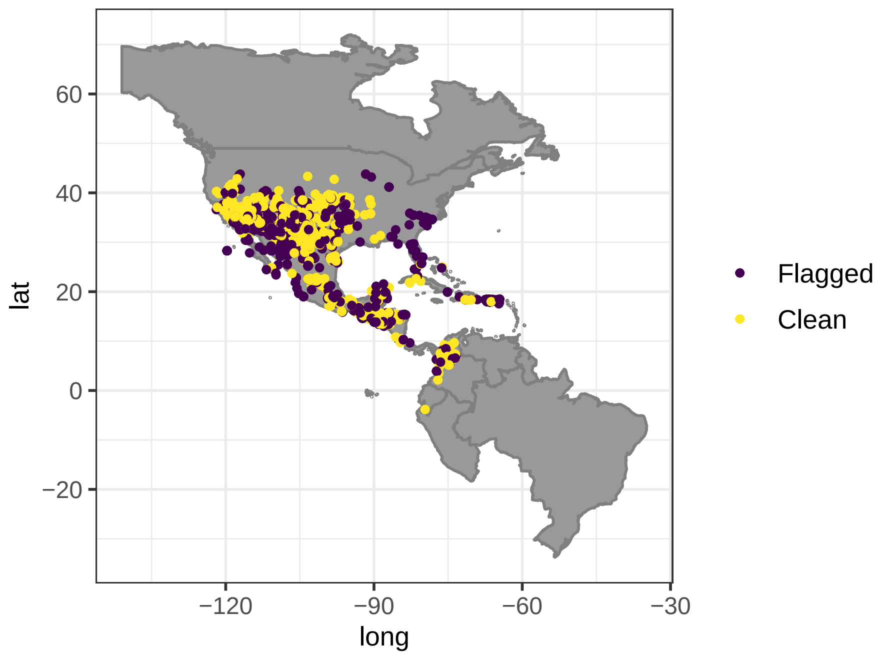


```
# Exclude problematic records
SA_teiines_occ_clean <- SA_teiines_occ[flags_SA$.summary,]
NA_teiines_occ_clean <- NA_teiines_occ[flags_NA$.summary,]
```

3.4 Download Environmental Variables

Download elevation data from Shuttle Radar Topography Mission, bioclimatic variables from WorldClim and NDVI data from Terra Satellite available in EarthData. The function `MODIStsp::MODIStsp()` requires a registration in EarthData and demands the user to provide an username and password to allow the download.

```
# Download elevation and bioclimatic variables
elevation <- geodata::elevation_global(res = 2.5,
                                       path = paste0(getwd(),"/WorldClim_data"))
bioclim <- geodata::worldclim_global(var = "bio",
                                     res = 2.5,
                                     path = paste0(getwd(),"/WorldClim_data"))

# Define areas
americas <- rnaturalearth::ne_countries(scale = "large",
                                        continent = c("South America",
```

```
                                                   "North America"),
                                        returnclass = "sf")
south_am <- rnaturalearth::ne_countries(scale = "large",
                                        continent = "South America",
                                        returnclass = "sf")
north_am <- rnaturalearth::ne_countries(scale = "large",
                                        continent = "North America",
                                        returnclass = "sf")

# Download NDVI data
#MODIStsp(gui = F,
#         out_folder = "VegetationData",
#         out_folder_mod = "VegetationData",
#         selprod = "Vegetation_Indexes_Monthly_005dg (M*D13C2)",
#         sensor = "Terra",
#         bandsel = "NDVI",
#         user = "user",
#         password = "password",
#         start_date = "2001.01.01",
#         end_date = "2020.12.31",
#         verbose = F,
#         spatmeth = "bbox",
#         bbox = c(-170,-56,-30,70),
#         out_format = "GTiff",
#         output_proj = "+proj=longlat +ellps=WGS84 +datum=WGS84 +no_defs",
#         delete_hdf = T,
#         parallel = detectCores() - 1)

# Convert all .tif files to layers in a SpatRaster
NDVI_files <- list.files("VegetationData/VI_Monthly_005dg_v6/NDVI",
                         full.names = T)


NDVI <- terra::rast(NDVI_files) %>%

  # Define projection
  terra::project(., "+proj=longlat +ellps=WGS84 +datum=WGS84 +no_defs") %>%

  # Calculate mean annual NDVI
  terra::app(., mean) %>%
```

```
  rename(., mean_NDVI = mean) %>%

  # Resample NDVI SpatRaster so its resolution matches the WorldClim data
  terra::resample(., bioclim, method = "bilinear")
```

```
|---------|---------|---------|---------|
=========================================

|---------|---------|---------|---------|
=========================================

|---------|---------|---------|---------|
=========================================
```

```
# Adjust scale
terra::scoff(NDVI) <- cbind(0.0001,0)

# Transform SpatRaster into a data frame
NDVI_df <- terra::as.data.frame(NDVI, xy = TRUE, na.rm = TRUE)
rownames(NDVI_df) <- c()

# Combine elevation, bioclimatic variables, and NDVI data
env <- c(elevation, bioclim, NDVI) %>%
  terra::mask(., NDVI) %>%
  terra::crop(., ext(-170,-30,-56,70))
```

```
|---------|---------|---------|---------|
=========================================

|---------|---------|---------|---------|
=========================================
```

```
# Crop to south and north america
env_SA <- terra::crop(env, south_am)
```

```
|---------|---------|---------|---------|
=========================================
```

```
env_NA <- terra::crop(env, north_am)
```

```
|---------|---------|---------|---------|
=========================================
```

3.5 Generate Pseudoabsences

```
# Generate pseudoabsences
pseudoaus_SA <- terra::spatSample(env_SA,
                                 size = nrow(SA_teiines_occ_clean),
                                 ext = south_am,
                                 xy = T,
                                 na.rm = T) %>%
                dplyr::select(., x, y)

pseudoaus_NA <- terra::spatSample(env_NA,
                                 size = nrow(NA_teiines_occ_clean),
                                 ext = north_am,
                                 xy = T,
                                 na.rm = T) %>%
                dplyr::select(., x, y)
```

3.6 Calculate Variance Inflation Factors (VIFs)

Remove highly collinear variables.

```
# Convert SpatRaster to data frame (takes some time)
env_df <- terra::as.data.frame(env, xy = T) %>%
  filter(complete.cases(.))

# Calculate VIFs
vifs <- usdm::vifstep(dplyr::select(env_df, -x, -y),
                      th = 3.5)
env_df <- usdm::exclude(env_df, vifs)
```

3.7 Extract Environmental Variable Data for Each Coordinate

```
# Extract Bioclimatic values for each coordinate
teiines_bioclim <- rbind(SA_teiines_occ_clean,
                         NA_teiines_occ_clean)
teiines_bioclim <- terra::extract(env,
                                  teiines_bioclim[,c("decimalLongitude",
                                                     "decimalLatitude")],
                                  xy = T,
                                  ID = F)



# Separate North and South America teiines and remove collinear variables
SA_teiines_bioclim <- teiines_bioclim %>%
  slice(1:nrow(SA_teiines_occ_clean)) %>%
  dplyr::select(x,
                y,
                vifs@results[["Variables"]]) %>%
  filter(., complete.cases(.)) %>%
  mutate(occurrenceStatus = "PRESENT")

NA_teiines_bioclim <- teiines_bioclim %>%
  slice(nrow(SA_teiines_occ_clean)+1:nrow(NA_teiines_occ_clean)) %>%
  dplyr::select(x,
                y,
                vifs@results[["Variables"]]) %>%
  filter(., complete.cases(.)) %>%
  mutate(occurrenceStatus = "PRESENT")

# Extract environmental variables for pseudoabsences
pseudoaus_SA_bioc <- terra::extract(env_SA,
                                    pseudoaus_SA[,c("x","y")],
                                    xy = T,
                                    ID = F) %>%
  mutate(occurrenceStatus = "PSEUDOABSENT") %>%
  dplyr::select(.,
                x,
                y,
                occurrenceStatus,
                vifs@results[["Variables"]])
```

```
pseudoaus_NA_bioc <- terra::extract(env_NA,
                                    pseudoaus_NA[,c("x","y")],
                                    xy = T,
                                    ID = F) %>%
  mutate(occurrenceStatus = "PSEUDOABSENT") %>%
  dplyr::select(.,
                x,
                y,
                occurrenceStatus,
                vifs@results[["Variables"]])

# Combine environmental data for presences and pseudoabsence
SA_teiines_pres_abs <- rbind(SA_teiines_bioclim, pseudoaus_SA_bioc)
NA_teiines_pres_abs <- rbind(NA_teiines_bioclim, pseudoaus_NA_bioc)
```

3.8 Ordinate Environmental Conditions

The correlation circle depicts the environmental variables used to characterize the ecological niche of North American teiids (*Aspidoscelis*, *Holcosus*, and *Pholidoscelis*) and their sister clade (*Ameiva*, *Cnemidophorus*, *Kentropyx*, and *Medopheos*). The angles between predictors correspond to the correlation between them and the projections of each vector on the axes are proportional to their contributions for each principal component.

```
# PCA
pca <- dudi.pca(rbind(SA_teiines_pres_abs, NA_teiines_pres_abs)[, 3:10],
                scannf = FALSE,
                nf = 2)

# Correlation circle
ecospat.plot.contrib(contrib = pca$co,
                     eigen = pca$eig)
```

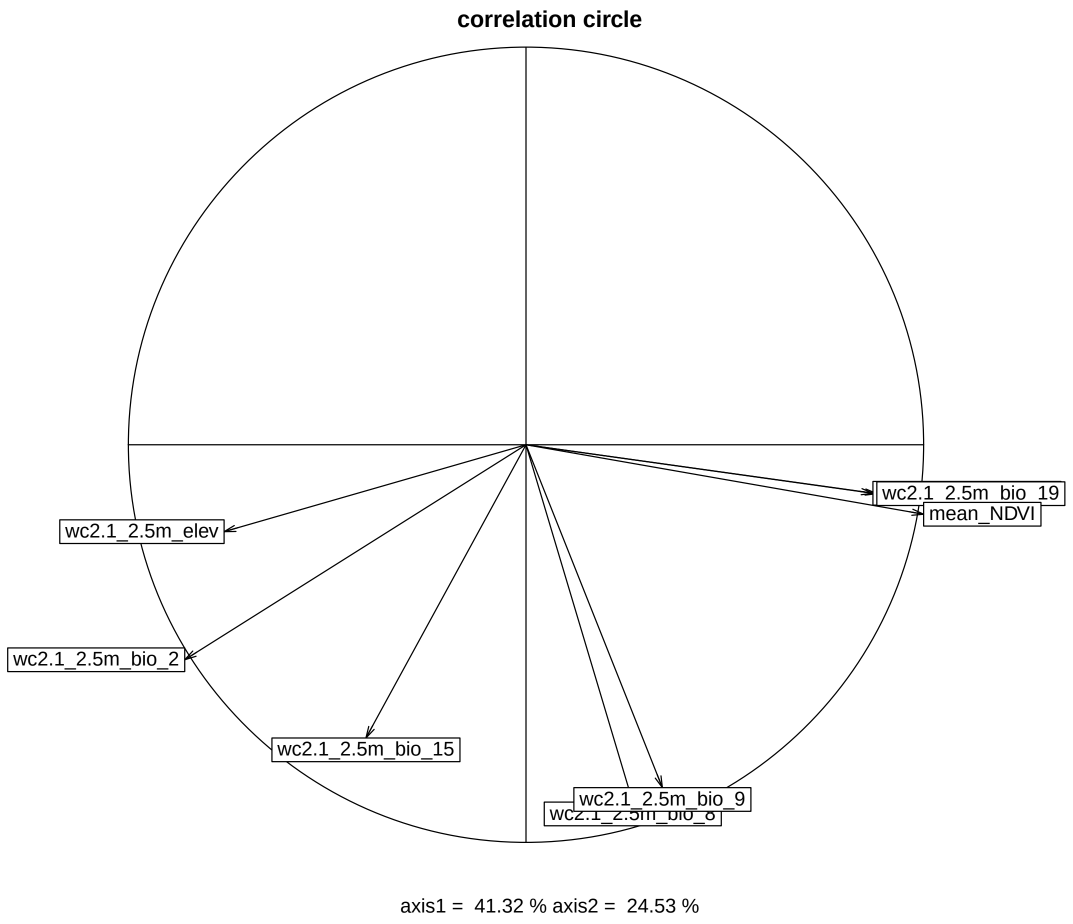

3.9 Calculate Ecological Niches in Native and Invaded Ranges

```
# Scores for all the New World (SA + NA)
scores_globclim <- pca$li

# Scores for the native area (SA)
scores_clim_nat <- suprow(pca,
                          SA_teiines_pres_abs[, 3:10])$li

# Scores for the invaded area (NA)
scores_clim_inv <- suprow(pca,
                          NA_teiines_pres_abs[, 3:10])$li
```

```
# Scores for the native distribution (SA where teiines occur)
scores_sp_nat <- suprow(pca,
                        SA_teiines_pres_abs[which(SA_teiines_pres_abs[,"occurrenceStatus"]
                            3:10])$li

# Scores for the invaded distribution (NA where teiines occur)
scores_sp_inv <- suprow(pca,
                        NA_teiines_pres_abs[which(NA_teiines_pres_abs[,"occurrenceStatus"]
                            3:10])$li


# Niche in the native distribution (SA)
grid_clim_nat <- ecospat.grid.clim.dyn(glob = scores_globclim,
                                       glob1 = scores_clim_nat,
                                       sp = scores_sp_nat,
                                       th.sp = 0)
```

```
Registered S3 methods overwritten by 'adehabitatMA':
  method                       from
  print.SpatialPixelsDataFrame sp
  print.SpatialPixels          sp
```

```
# Niche in the invaded distribution (NA)
grid_clim_inv <- ecospat.grid.clim.dyn(glob = scores_globclim,
                                       glob1 = scores_clim_inv,
                                       sp = scores_sp_inv,
                                       th.sp = 0)
```

3.10 Niche Equivalency and Similarity Tests

Test two hypothesis (equivalency/similarity higher than random or lower than random) for both niche equivalency and niche similarity.

```
# Niche equivalency test
eq_test_h <- ecospat.niche.equivalency.test(grid_clim_nat,
                                            grid_clim_inv,
                                            rep = 1000,
                                            overlap.alternative = "higher",
                                            ncores = detectCores() - 1)
```

```r
eq_test_l <- ecospat.niche.equivalency.test(grid_clim_nat,
                                            grid_clim_inv,
                                            rep = 1000,
                                            overlap.alternative = "lower",
                                            ncores = detectCores() - 1)

# Significance
eq_test_h$p.D
```

```
[1] 1
```

```r
eq_test_l$p.D
```

```
[1] 0.000999001
```

```r
# Niche similarity test (randomization of the niche in the invaded area)
sim_test_h <- ecospat.niche.similarity.test(grid_clim_nat,
                                          grid_clim_inv,
                                          rep = 1000,
                                          overlap.alternative = "higher",
                                          rand.type = 1,
                                          ncores = detectCores() - 1)

sim_test_l <- ecospat.niche.similarity.test(grid_clim_nat,
                                            grid_clim_inv,
                                            rep = 1000,
                                            overlap.alternative = "lower",
                                            rand.type = 1,
                                            ncores = detectCores() - 1)
# Significance
sim_test_h$p.D
```

```
[1] 0.1178821
```

```r
sim_test_l$p.D
```

```
[1] 0.8891109
```

3.11 Niche Equivalency/Similarity Histograms

Histograms of Schoener's D values for 1000 simulations in each test. The red lines show the observed Schoener's D value. The title informs the type of test (equivalency/similarity) and the alternative hypothesis (greater than random/lower than random).

```r
# Niche Similarity graph
par(mfrow = c(2,2))

ecospat.plot.overlap.test(eq_test_h,
                          "D",
                          "Equivalency (greater)")

ecospat.plot.overlap.test(eq_test_l,
                          "D",
                          "Equivalency (lower)")

ecospat.plot.overlap.test(sim_test_h,
                          "D",
                          "Similarity (greater)")

ecospat.plot.overlap.test(sim_test_l,
                          "D",
                          "Similarity (lower)")
```

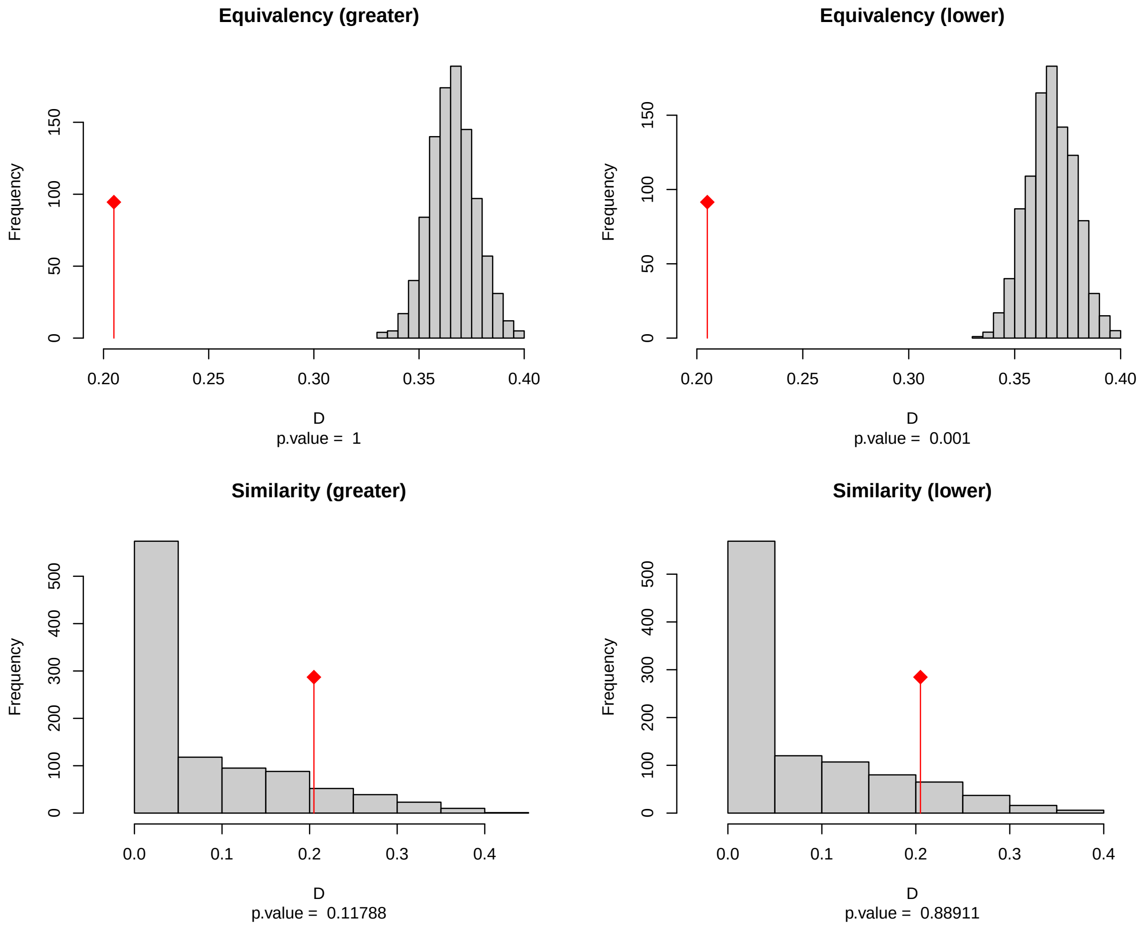

3.12 Niche Overlap Graph

Niche overlap between North American teiids (*Aspidoscelis*, *Holcosus*, and *Pholidoscelis*) and their sister clade (*Ameiva*, *Cnemidophorus*, *Kentropyx*, and *Medopheos*). The axes depict the principal components of the environmental variables used to characterize the ecological niche. The lines represent the ecological conditions in North America (red) and South America (green). The blue stain corresponds to the niche overlap between both lineages, the red stain corresponds to the ecological niche exclusive to the northern lineage and the green stain corresponds to the ecological niche exclusive to the southern lineage. The shading indicates the density of registers of the northern clade.

```
# Niche overlap graph
ecospat.plot.niche.dyn(grid_clim_nat,
                       grid_clim_inv,
```

```
interest = 2,
name.axis1 = "PC1",
name.axis2 = "PC2",
col.unf = "green",
col.exp = "red",
col.stab = "blue",
colZ1 = "green",
colZ2 = "red")
```

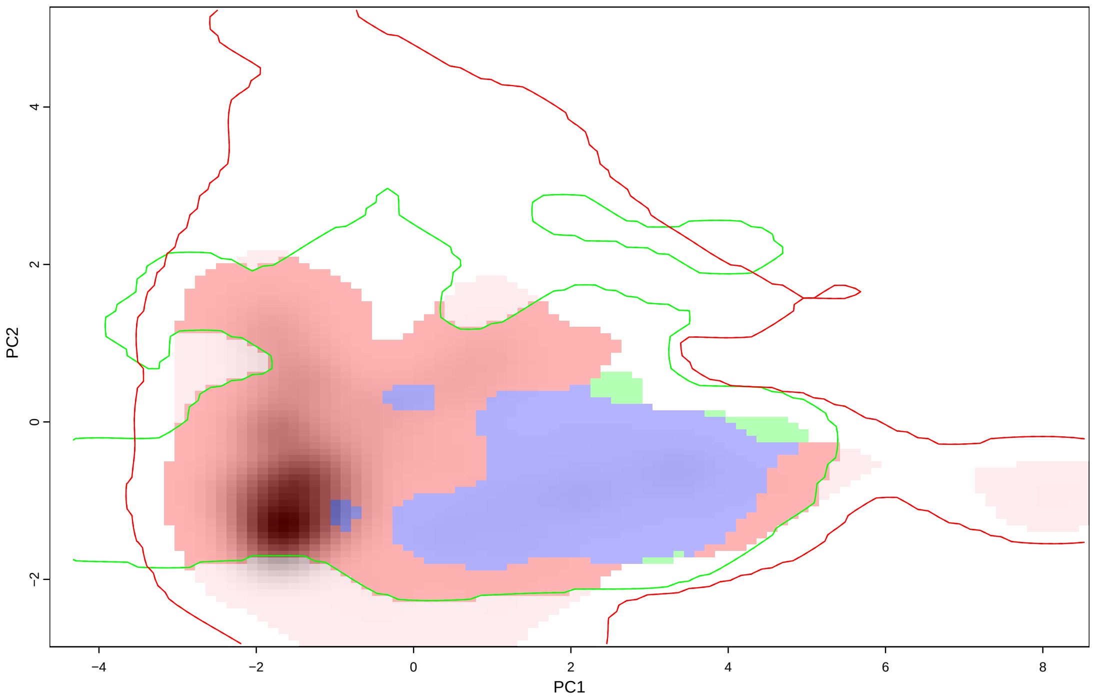

Supplement: Supplementary file 1 — Data S1. [file ECE3-14-e70418-s001.zip › ece370418-sup-0006-FileS5.pdf]
